# Cumulative meta-analysis of interleukins 6 and 1β, tumour necrosis factor α and C-reactive protein in patients with major depressive disorder

**DOI:** 10.1016/j.bbi.2015.06.001

**Published:** 2015-10

**Authors:** Rita Haapakoski, Julia Mathieu, Klaus P. Ebmeier, Harri Alenius, Mika Kivimäki

**Affiliations:** a Department of Epidemiology and Public Health, University College London, London, UK; b Finnish Institute of Occupational Health, Systems Toxicology Unit, Centre of Expertise for Health and Work Ability, Helsinki, Finland; c Department of Psychiatry, University of Oxford, Warneford Hospital, Oxford, UK; d Université Pierre et Marie Curie, Paris, France; e Department of Public Health, Faculty of Medicine, University of Helsinki, Helsinki, Finland

**Keywords:** Major depression, Inflammation, Interleukin-6, Interleukin-1β, Tumour necrosis factor-α, C-reactive protein, Cumulative meta-analysis

## Abstract

Cumulative meta-analyses are used to evaluate the extent to which further studies are needed to confirm or refute a hypothesis. We used this approach to assess observational evidence on systemic inflammation in individuals with major depressive disorder. We identified 58 studies of four common inflammatory markers in a literature search of PubMed, Embase and PsychInfo databases in May 2014. Pooled data from the earliest eight studies already showed an association between interleukin-6 concentrations and major depression; 23 more recent studies confirmed this finding (*d* = 0.54, *p* < 0.0001). A significant association between C-reactive protein levels and major depression was noted after 14 studies and this did not change after addition of six more studies (*d* = 0.47, *p* < 0.0001). For these two inflammatory markers, there was moderate heterogeneity in study-specific estimates, subgroup differences were small, and publication bias appeared to be an unlikely explanation for the findings. Sensitivity analyses including only high-quality studies and subjects free of antidepressant medication further verified the associations. While there was a link between tumour necrosis factor-α levels and major depression (*d* = 0.40, *p* = 0.002), the cumulative effect remained uncertain due to the extensive heterogeneity in study-specific estimates and inconsistencies between subgroups. No evidence was found for the association between interleukin-1β levels and major depression (*d* = −0.05, *p* = 0.86). In conclusion, this cumulative meta-analysis confirmed higher mean levels of interleukin-6 and C-reactive protein in patients with major depression compared to non-depressed controls. No consistent association between tumour necrosis factor-α, interleukin-1β and major depression was observed. Future studies should clarify the specific immune mechanisms involved as well as continue testing anti-inflammatory therapies in patients suffering from major depression.

## Highlights

• This meta-analysis confirms a robust link between IL-6, CRP and major depression.
• The role of TNF-α and IL-1β in major depression remains uncertain.
• Further mechanistic and immunotherapeutic studies on IL-6 and CRP are needed.

## 1 Introduction

Major depressive disorder (MDD) is a leading cause of disability and an important contributing factor to the worldwide burden of disease; it can directly cause mortality due to suicide and indirectly through affecting prognosis of chronic conditions such as ischemic heart disease (Whiteford et al., 2013). The inflammation hypothesis of depression proposes that MDD is also associated with sustained activation of the innate immune system leading to increased production of proinflammatory cytokines and acute phase proteins (Maes, 1995; Smith, 1991). In support of this hypothesis, several studies have detected higher levels of inflammation in depressed individuals than in healthy controls, although the strength of evidence varies depending on which specific inflammatory markers have been examined, i.e. interleukin (IL)-6, IL-1β, tumour necrosis factor (TNF)-α and C-reactive protein (CRP) (Dowlati et al., 2010; Hiles et al., 2012b; Howren et al., 2009; Liu et al., 2012; Valkanova et al., 2013).

In a cumulative meta-analysis, the results from individual studies are added chronologically and the aggregate effect estimate recomputed after addition of each study. The purpose of this technique is to determine the point at which further studies carried out in a similar manner are unlikely to change the conclusion. Importantly, information in these analyses may help researchers to justify additional studies or to become aware of the need for redirecting their research efforts (Clarke et al., 2014). Here, we have applied this approach to evaluate the strength of the association between specific inflammatory markers (IL-6, IL-1β, CRP-α, TNF) and MDD.

## 2 Materials and methods

### 2.1 Literature search and data extraction

We performed a systematic search of the literature using PubMed, Embase, PsychInfo to include publications until May 2014 with the following search terms: [“*depression*”], [“*depressive*”], [“*C-reactive protein*”], [“*CRP*”], [“*interleukin 6*”], [“*IL-6*”], [“*tumour necrosis factor α*”], [*TNF-alpha*”], [“*interleukin 1β*”], [*IL-1beta*”], [“*cytokine*”]. We also scrutinised the reference lists of all of the relevant publications identified. In addition, we used the Institute of Scientific Information Web of Science (May 2014) to retrieve all the studies citing the studies identified by the search. Information from the articles included in the meta-analysis reporting an association between CRP (Howren et al., 2009), IL-6, TNF-α, IL-1β (Dowlati et al., 2010; Hiles et al., 2012b; Howren et al., 2009; Liu et al., 2012) and depression in cross-sectional settings was reviewed and retracted. Data were searched and extracted independently by two authors (RH and JM), the results were compared and consensus obtained through discussion. Supplementary Fig. 1 shows the literature search strategy.

### 2.2 Selection criteria

The immune markers evaluated in this study were selected based on their effects on the development of sickness behaviour (IL-1β and TNF-α) (Dantzer, 2001; Dantzer et al., 2008) and previous meta-analyses suggesting possible associations of peripheral levels of IL-1, (Howren et al., 2009) TNF-α, (Dowlati et al., 2010; Liu et al., 2012) and IL-6, (Dowlati et al., 2010; Hiles et al., 2012b; Liu et al., 2012) CRP (Howren et al., 2009) with depression. Previous meta-analyses did not support the involvement of IL-10, (Dowlati et al., 2010; Hiles et al., 2012b; Liu et al., 2012) IL-4, (Dowlati et al., 2010; Liu et al., 2012) IL-8, (Dowlati et al., 2010; Liu et al., 2012) IL-2, (Dowlati et al., 2010; Liu et al., 2012) or IFN-γ (Dowlati et al., 2010; Liu et al., 2012) in depression, and therefore these cytokines were not included in this analysis.

Specific inclusion criteria were (1) measurement of unstimulated cytokines from the blood; (2) comparison of adult patients with MDD and psychiatrically healthy controls; (3) use of structured clinical assessments (SCID or MINI) based on DSM-III/DSM-IV criteria to verify unipolar MDD; (4) incorporation of study subjects without major physical illness (i.e. diabetes, heart disease, cancer etc.) at the time of assessment. Studies focusing on pregnant women, postpartum depression or including patients with metabolic syndrome were excluded from the meta-analysis, whereas studies targeting patients with stable medical conditions, such as hypertension were included. Studies where participants used any medications at the time of blood sampling were referred to as “*subjects using medication*”. Individuals that had undergone a washout period from antidepressants before entering the study or at the time of immune assessments were referred to as “*subjects not using antidepressant medication*”. The median washout period in the included studies was 4 weeks with a range between 1 week and 3 months (Supplementary Table 3).

### 2.3 Quality assessment

This meta-analysis was conducted according to PRISMA guidelines (Moher et al., 2009). For evaluation of the quality of included studies, we used the Newcastle-Ottawa quality assessment scale (NOS) for case-control studies (Wells et al., 2009) In this method, each study can obtain a maximum of 9 points in three categories; selection of study participants (adequate definition, validation and representativeness of cases and controls), comparability of cases and controls, and the ascertainment of exposure (Supplementary Table 1). In line with the recommendations of the Meta-Analysis of Observational Studies, (Stroup et al., 2000), we performed a sensitivity analysis excluding those studies with poor quality (NOS score < 6). We also performed an additional quality analysis using the score of 7 as the cut-off value.

### 2.4 Data synthesis

The statistical package Comprehensive Meta-analysis (version 2) was used for data analysis. Effect sizes were calculated using standardised mean differences (Cohen’s *d*) from mean (±SD) concentrations of each immune marker measured in the blood of MDD patients and non-depressed controls. If concentrations of immune markers were not reported, we extracted the effect size from exact *p*-values and sample size (Dahl et al., 2014; Euteneuer et al., 2011; Hornig et al., 1998; Li et al., 2013). When relevant data were not reported, (Grassi-Oliveira et al., 2009; Maes et al., 1995b; Weinstein et al., 2010; Yang et al., 2007) we first contacted the authors for assistance and if not successful, retrieved values from graphs using DataThief III, version 1.6. (Tummers et al., 2008).

A cumulative meta-analysis was performed by sorting of the included studies according to the date of publication. The *p*-values represent the summary statistics of all incorporated studies (rather than *p*-values for independent studies), enabling the inspection of the contribution of each new study to the overall effect estimate. Due to the variation in effect sizes across studies and to achieve generalisation of the results, a random-effects model was used in the analysis of total data and in the meta-regression. Random-effects models take into account both within- and between-study variability, and provide a more conservative estimate of effect sizes than can be obtained with the fixed-effect models. Heterogeneity across studies was assessed using Cochrane’s *Q* and *p* < 0.10 was considered as significant. The *I*^2^ index < 25% was defined as ‘low’, 50% as ‘moderate’ and 75% ‘high’ heterogeneity (Higgins et al., 2003). Publication bias was examined using Egger’s regression intercept and Begg and Mazumdar’s rank correlation. We used four criteria to determine whether further research conducted in the same manner is likely to be needed to confirm the conclusion: (1) the association has reached statistical significance several years ago and the effect size has remained unchanged after the addition of more recent studies; (2) in the total evidence, there is very little heterogeneity in study-specific estimates; (3) any subgroup differences are small; and (4) publication bias is an unlikely explanation for the findings.

Potential covariates were selected, based on earlier reports showing associations of age, gender and BMI with both inflammatory markers and depression (Hiles et al., 2012b; Howren et al., 2009; Liu et al., 2012). When data were presented separately for different subgroups of patients, we computed a combined effect size for final analysis. Mean age of study population (>40 vs. ⩽40 years), gender (>50% vs. ⩽50% of study subjects female), sample size (over 50 subjects was defined as large), sample quality (⩾6 was defined as high quality), patient type (inpatient vs. outpatient) and BMI (controlled for vs. not controlled) were used as categorical effect modifiers in the subgroup analyses. In the sensitivity analysis, we performed meta-analyses using only high quality studies according to two different cut-off scores (NOS ⩾ 6 and ⩾ 7) and studies including only participants not using antidepressants at the time of blood sampling.

## 3 Results

After excluding duplicates, reviews, animal studies and non-relevant studies, the full texts of remaining articles (*n* = 209) were inspected in detail (Supplementary Fig. 1). Articles were excluded when a diagnosis of MDD was not made according to Diagnostic and Statistical Manual of Mental Disorders (DSM) criteria and when studies did not employ sufficiently exact and specific patient exclusion criteria (*N* = 93). We excluded studies which had included patients with minor depression (*N* = 1) and bipolar disorder (*N* = 2) in addition to MDD patients. Studies were also excluded if patients suffered from a significant comorbid disease (*N* = 31), or if the study lacked a proper control group (*N* = 16), if inflammatory marker measurements had been assayed in fluids other than venous blood (*N* = 5) or if *in vitro* stimulated cytokines had been used to assess immune function (*N* = 3). In total, 58 articles met the inclusion criteria: 20 studies for CRP (Cizza et al., 2009; Dome et al., 2009; Frodl et al., 2012; Häfner et al., 2008; Hornig et al., 1998; Hughes et al., 2012; Joyce et al., 1992; Karlovic et al., 2012; Keri et al., 2014; Kling et al., 2007; Lanquillon et al., 2000; O’Donovan et al., 2013; Piletz et al., 2009; Rothermundt et al., 2001; Rudolf et al., 2014; Sluzewska et al., 1996; Thomas et al., 2005; Tuglu et al., 2003; Weinstein et al., 2010; Zahn et al., 2013), 31 studies for IL-6 (Basterzi et al., 2005; Carvalho et al., 2013; Dahl et al., 2014; Dhabhar et al., 2009; Dunjic-Kostic et al., 2013; Euteneuer et al., 2011; Fitzgerald et al., 2006; Fornaro et al., 2011; Frodl et al., 2012; Hennings et al., 2013; Hughes et al., 2012; Kagaya et al., 2001; Karlovic et al., 2012; Keri et al., 2014; Leo et al., 2006; Maes et al., 1997, 1995a,b; Mikova et al., 2001; Motivala et al., 2005; O’Brien et al., 2007; O’Donovan et al., 2013; Pavon et al., 2006; Pike and Irwin, 2006; Rudolf et al., 2014; Simon et al., 2008; Sluzewska et al., 1996; Voderholzer et al., 2012; Weinstein et al., 2010; Yang et al., 2007; Yoshimura et al., 2010), 31 studies for TNF-α (Dahl et al., 2014; Diniz et al., 2010b; Dome et al., 2009; Dunjic-Kostic et al., 2013; Eller et al., 2009, 2008; Euteneuer et al., 2011; Fitzgerald et al., 2006; Fornaro et al., 2013; Grassi-Oliveira et al., 2009; Hornig et al., 1998; Huang and Lee, 2007; Hughes et al., 2012; Kagaya et al., 2001; Karlovic et al., 2012; Leo et al., 2006; Li et al., 2013; Maes et al., 2012a,b; Mikova et al., 2001; Narita et al., 2006; O’Brien et al., 2007; O’Donovan et al., 2013; Pavon et al., 2006; Piletz et al., 2009; Schmidt et al., 2014; Simon et al., 2008; Sutcigil et al., 2007; Tuglu et al., 2003; Weinstein et al., 2010; Yang et al., 2007) and 14 studies for IL-1β (Dahl et al., 2014; Diniz et al., 2010a; Fornaro et al., 2013; Hernandez et al., 2008, 2013; Huang and Lee, 2007; Hughes et al., 2012; Kagaya et al., 2001; Leo et al., 2006; Pavon et al., 2006; Piletz et al., 2009; Simon et al., 2008; Thomas et al., 2005; Yang et al., 2007). Supplementary Table 2 presents the descriptive and quality data of each study and Supplementary Table 3 shows the specific inclusion and exclusion criteria used in these studies.

### 3.1 Cumulative meta-analyses

#### 3.1.1 Interleukin 6

**Fig. 1:**
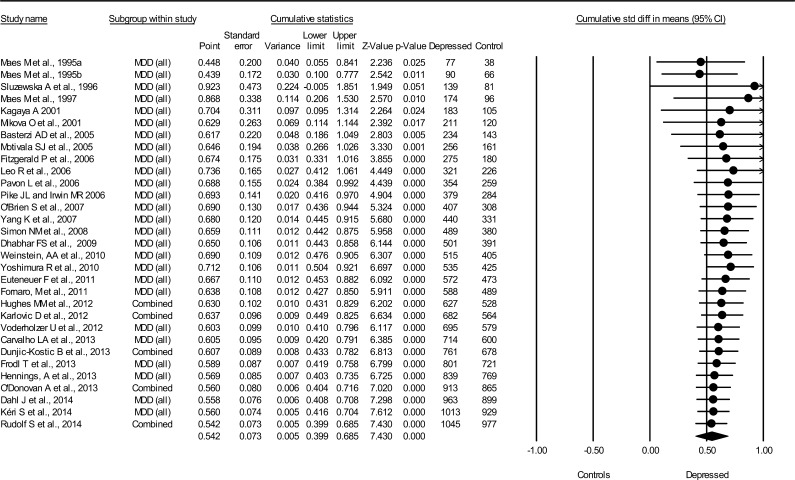
Cumulative meta-analysis for IL-6 levels and major depressive disorder.

The cumulative meta-analysis showed a medium-sized association between IL-6 and MDD (*N* = 31, combined *d* = 0.54, 95% CI = 0.40–0.69, total *N*(MDD) = 1045, total *N*(non-MDD) = 977) (Fig. 1). A statistically significant association (*p* < 0.0001) was first achieved in 2006, and this result has remained unchanged after 23 more studies published between the years 2006 and 2014. Moderate heterogeneity between studies was detected (*Q* = 68.5, *p* < 0.0001, *I*^2^ = 56.2%), but there was no apparent publication bias: Begg and Mazumdar’s tau 0.16 (*p* = 0.21), Egger’s regression intercept 1.13 (*p* = 0.30).

#### 3.1.2 C-reactive protein

**Fig. 2:**
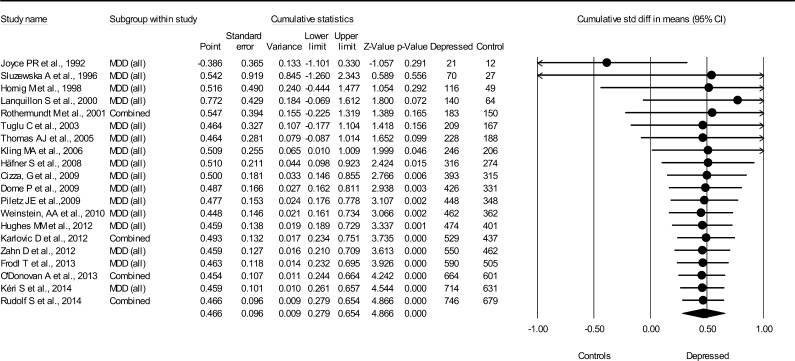
Cumulative meta-analysis for CRP levels and major depressive disorder.

There was a medium-sized association between CRP and MDD (*N* = 20, combined *d* = 0.47; 95% CI = 0.28–0.65, total *N*(MDD) = 746, total *N*(non-MDD) = 679) (Fig. 2). Statistical significance (*p* < 0.0001) was achieved after 14 studies, and the association did not change after supplementing with six more studies. Heterogeneity between studies was moderate (*Q* = 51, *p* < 0.0001, *I*^2^ = 62%) and no apparent publication bias was notified (Begg and Mazumdar’s tau 0.06 (*p* = 0.72), Egger’s regression intercept 1.02 (*p* = 0.47).

#### 3.1.3 Tumour necrosis factor α

**Fig. 3:**
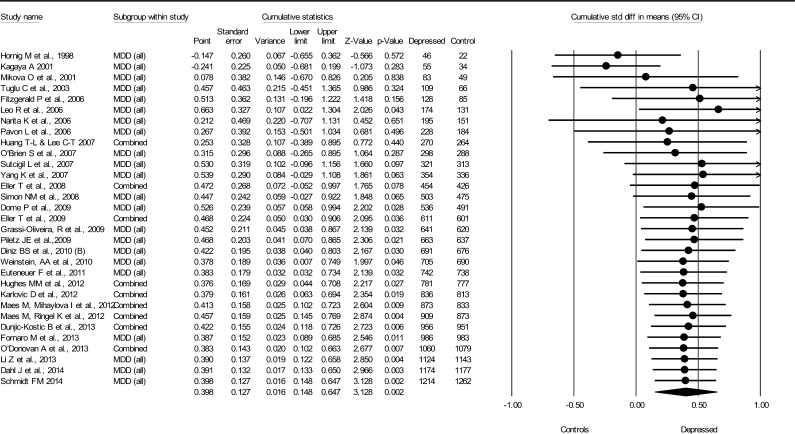
Cumulative meta-analysis for TNF-α levels and major depressive disorder.

The pooled evidence showed increased blood levels of TNF-α in MDD patients (*N* = 31, combined *d* = 0.40, 95% CI = 0.15–0.65, total *N*(MDD) = 1214, total *N*(non-MDD) = 1262) (Fig. 3). The effect reached statistical significance (*p* < 0.05) after 14 studies in 2009 and the size of the association did not substantially change after the publication of 17 additional articles. Heterogeneity in study-specific effect estimates was high (*Q* = 254, *p* < 0.0001, *I*^2^ = 88%), suggesting that systematic differences existed between studies. There was no apparent publication bias (Begg and Mazumdar’s tau 0.22 (*p* = 0.08), Egger’s regression intercept 2.91 (*p* = 0.12).

#### 3.1.4 Interleukin 1β

**Fig. 4:**
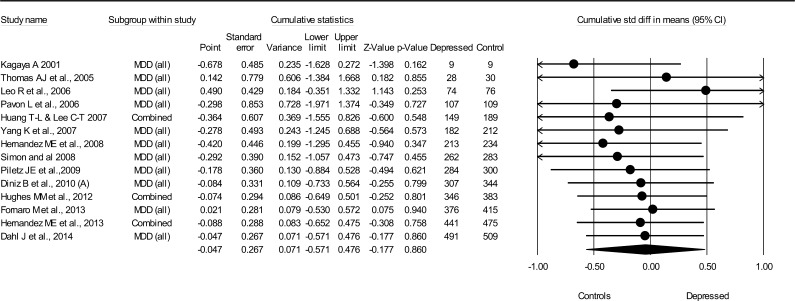
Cumulative meta-analysis for IL-1β levels and major depressive disorder.

IL-1β was not associated with MDD (*N* = 14, combined *d* for all studies = −0.05, 95%CI = −0.57–0.48, total *N*(MDD) = 491, total *N*(non-MDD) = 509) (Fig. 4). There was extensive heterogeneity between studies (*Q* = 197, *p* < 0.0001, *I*^2^ = 93%), but no publication bias (Begg and Mazumdar’s tau −0.09 (*p* = 0.66), Egger’s regression intercept was −0.25 (*p* = 0.96).

### 3.2 Additional analyses

#### 3.2.1 Sensitivity analyses

When only high quality studies (NOS-score ⩾ 6) were included in the analysis, the association between IL-6 and MDD remained statistically significant and stable after completion of the first five studies (*N* = 21 studies, *d* = 0.60, 95%CI = 0.42–0.78, total *N*(MDD) = 781, total *N*(non-MDD) = 711, *p* < 0.0001) (Table 1). Including only subjects not using antidepressants decreased the total number of studies to 16, but the association remained strong and statistically significant after the publication of 11 recent studies (*d* = 0.65, *p* < 0.0001) (Supplementary Fig. 2A). Using the alternative cut-off score ⩾7 for high quality revealed that the association had remained significant and unaltered since the publication of the first four studies (*N* = 8, *d* = 0.65, *p* < 0.0001).

With respect to CRP, exclusion of lower quality studies (NOS-score < 6) confirmed the positive association with MDD: *N* = 10, *d* = 0.69, 95%CI = 0.43–0.95, total *N*(MDD) = 395, total *N*(non-MDD) = 321) (Table 1). Further exclusions of the studies allowing the use of medications during the blood draw sampling led to even increased effect size estimates (*d* = 0.88, *p* < 0.0001). These associations have remained unaltered since 1996 to 2014 after completion of seven additional studies (Supplementary Fig. 2B). The use of the cut-off score ⩾7 for quality assessment left only five studies for analysis; however, the association still remained statistically significant (*d* = 0.69, *p* = 0.002).

In the analysis of high quality studies, the association between TNF-α and MDD weakened and changed to being statistically non-significant (*N* = 18, *d* = 0.28, *p* = 0.09, 95%CI = −0.04–0.59 total *N*(MDD) = 805, total *N*(non-MDD) = 872) (Table 1). Further exclusion of studies permitting the concomitant use of medications resulted in a positive but statistically unstable association between TNF-α and MDD (*N* = 12, *d* = 0.57, *p* = 0.004) (Supplementary Fig. 2C).

Our sensitivity analysis confirmed the lack of association between IL-1β and MDD in the high quality (NOS > 6) studies restricted to patients free of antidepressant medication (*N* = 9, *d* = −0.36, *p* = 0.29) (Supplementary Fig. 2D).

#### 3.2.2 Subgroup differences

Age, gender, BMI, medication use, study size or patient type did not modify the association between IL-6 and MDD (Table 1). TNF-α was significantly related to MDD in studies restricted to antidepressant-free subjects (*p* = 0.001), studies including younger participants (age < 40) (*p* = 0.001), as well as those not controlling for BMI (*p* = 0.006) and including more female than male study subjects (*p* = 0.004). The association between IL-1β and MDD was statistically significant only in older patients (*p* < 0.0001).

Majority of studies (*N* = 38, 66%) reported depressive symptoms being severe or very severe (Supplementary Table 4). Severe depression was more strongly associated with IL-6, CRP and TNF-α compared to moderate depression (Table 1).

A meta-regression revealed that a higher mean age was associated with weaker associations between TNF-α and MDD (*p* = 0.01), and stronger associations between IL-1β and MDD (*p* = 0.007). There was a suggestive trend towards stronger associations between IL-6 and MDD with increased mean age (*p* = 0.06). No significant associations between immune markers and HAMD score (an indicator of the severity of symptoms) were found.

#### 3.2.3 Other Axis I/II disorders and depression subtypes

Information on other Axis I or Axis II diagnoses, such as generalised anxiety disorders or any anxiety or psychotic disorders, panic disorder, phobia, obsessive–compulsive disorder, post-traumatic stress disorder, schizophrenia or bipolar disorder, had been included in 27 (47%) of the 58 studies (Supplementary Table 4). Fifteen (26%) studies did not assess or report other Axis I or Axis II disorders and 26% excluded all patients with any other Axis I or Axis II disorder other than MDD. Thirteen studies (22%) excluded patients with psychotic features, 10 (17%) with bipolar disorder and 4 (7%) with comorbid anxiety symptoms.

Most studies (72%) did not include information on the different subtypes of MDD. MDD with melancholic features had been investigated in 10 (17%) studies, atypical and suicidal/non-suicidal MDD in 3 (5%) and treatment-resistant MDD in 5 (9%) studies. Given the large variability in study-specific inclusion and exclusion criteria for other Axis I/II disorders and a small number of studies specifying different depression subtypes, we could not assess the role of these covariates on the depression-inflammation relation.

#### 3.2.4 Pharmacotherapy

From the 39 studies not allowing antidepressant medication during the inflammatory marker assessments, 35 (90%) specified the use of SSRIs and/or other classes of antidepressants as an exclusion criterion and four studies (10%) used the term “medication free” as their inclusion criterion (Supplementary Table 4). Fourteen studies (36%) had specified the concomitant use of antidepressants and other psychotropic medications (including anxiolytics, antipsychotics and/or mood stabilizers) as exclusion criteria and 14 (36%) excluded patients using both antidepressant and anti-inflammatory medications.

#### 3.2.5 Lifestyle factors

Twenty-two (38%) studies included smokers, 16 (28%) studies did not give information on substance use (smoking, drinking or drug use) and 24 (41%) had excluded patients with drug abuse or substance dependence (Supplementary Table 4). Since only 6 (10%) of studies had excluded subjects who were smokers, the role of smoking on inflammation–depression associations could not be verified. In the high-quality studies with subjects not using antidepressants, 12/16 (75%) for IL-6 and 5 of 7 studies (71%) for CRP had excluded patients with previous/current substance abuse.

#### 3.2.6 Other potential covariates

Other covariates available in some of the studies, such as the number of depression episodes and the duration and age of onset of depressive disorder (Supplementary Table 3), were not considered in the current meta-analysis. The effect of physical comorbidity on the inflammatory marker–depression association could not be tested given the nature of the available information.

## 4 Discussion

Using cumulative meta-analysis, we sought to determine the strength of the totality of evidence and the year when the association between specific immune markers and major depressive disorder first reached statistical significance. In addition, we explored the effect of subsequent studies on the overall effect size enabling us to evaluate whether new studies are likely to change these associations (Clarke et al., 2014). We found strong support for higher circulating IL-6 concentrations in patients with major depression in comparison to non-depressive individuals. This association has remained essentially unchanged from 2006 until the present date and including 23 additional studies. Similarly, the CRP–major depression relationship is robust and has been unaltered since 2012 after addition of six more recent publications. Nonetheless, these additional data, while having only a marginal effect on the overall effect size estimate, have narrowed the 95% confidence intervals. Additional sensitivity analyses incorporating only high-quality studies of subjects not receiving antidepressant drug treatment further strengthened these relationships, evidence that the observed associations are robust and unlikely to change should new studies be conducted using similar designs.

Our findings on IL-6 and CRP are in agreement with preclinical data showing a plausible role of IL-6 in the pathology of depressive-like behaviour (Chourbaji et al., 2006; Sukoff Rizzo et al., 2012), predictive associations of IL-6 and CRP with the future development of mood disorders (Gimeno et al., 2009; Khandaker et al., 2014; Kivimaki et al., 2014; Virtanen et al., 2015; Wium-Andersen et al., 2013), evidence of dose–response relationships (Kivimaki et al., 2014) and associations of these inflammatory markers with therapeutic response to antidepressants and ketamine (Cattaneo et al., 2013; Dahl et al., 2014; Hiles et al., 2012a; Yang et al., 2014). Our findings also confirm those reported in previous meta-analyses which included a smaller number of cross-sectional (Dowlati et al., 2010; Liu et al., 2012) and longitudinal studies (Valkanova et al., 2013) on IL-6 and CRP. Subgroup analysis showed that the associations of IL-6 and CRP concentrations with major depression remained relatively stable when assessing the effect of age, gender, BMI, study size or study quality on overall effect estimate.

The association between TNF-α and major depression, although the volume of existing research for this inflammation marker is equal to or even greater than that examining IL-6 or CRP, was less convincing: the overall effect size was smaller, the findings in sensitivity and subgroup analyses were less consistent and, importantly, the association was attenuated in higher quality studies and studies controlling for obesity. These results may result from a weaker association, failure to properly control for confounding factors (Hiles et al., 2012b) or difficulties in measuring accurately this inflammatory marker. In all these cases, improvements in research protocols and study designs, rather than simply more research conducted in a similar manner, are needed. Furthermore, the divergent effects of anti-TNF-α therapy on depressive symptoms (Kohler et al., 2014) point to the existence of potential confounders moderating the strength of the association between TNF-α and major depression.

Aggregate data from the 15 studies on IL-1β did not convincingly confirm or refute an association with major depression. The summary effect estimate based on these 15 studies was completely null, whereas for CPR and IL-6, the summary estimate had reached statistical significance after the completion of 14 or 8 studies, respectively. Elevated IL-1β levels in subjects with major depression, in comparison to non-depressive controls, were observed only in studies including subjects older than 40 years of age and studies rated as lower quality. The lack of a robust association between IL-1β and major depression might be partly attributable to measurement issues, as the concentrations of IL-1β are very low in blood and therefore more difficult to determine accurately using conventional immunological assays. Further research on a range of IL-1-family proteins (e.g. IL1Ra, soluble ILRII or soluble ST2), in addition to IL-1β, could increase our understanding of the role of IL-1 in major depression.

In general, the strength of the association between inflammation and major depression may vary depending on methods of depression diagnosis, sample characteristics, and control for confounders. For example, the relationships between venous blood levels of IL-6, CRP and depression have been weaker in studies using self-reported instruments rather than clinical diagnosis to ascertain depression, in community-based subjects compared to clinical samples, and when participants treated with antidepressants at the time of immune assessment have been included in the analysis (Hannestad et al., 2011; Hiles et al., 2012a,b; Howren et al., 2009). Furthermore, it is plausible that different subtypes of depressive disorder may be associated with different inflammatory profiles (Dunjic-Kostic et al., 2013; Kaestner et al., 2005; Karlovic et al., 2012). Similarly, chronic diseases, severity of depressive symptoms and comorbid psychiatric disorders may affect the magnitude of the associations between inflammation and major depression (Hiles et al., 2012b; Howren et al., 2009; Irwin and Miller, 2007; Stuart and Baune, 2012; Van der Kooy et al., 2007). The majority of major depressive patients included in our meta-analysis could be classified as severely or very severely depressed; IL-6, CRP and TNF-α were more strongly associated with severe than non-severe forms of depression. In an attempt to minimise the effect of chronic diseases and medications on immune-depression associations, we only included patients free of major chronic diseases and antidepressant use at the time of blood sampling in our sensitivity analysis. These analyses revealed that the association between IL-6, CRP and major depression remained strong and unaltered in studies of higher quality and antidepressant-free patients.

Compared to the most recent meta-analyses on this topic (Dowlati et al., 2010; Liu et al., 2012), our cumulative meta-analysis includes seven additional articles on IL-6 and eight on TNF-α that employed subjects not on antidepressants treatment and free of major physical diseases. We also included 10 additional studies on IL-6 and 13 on TNF-α allowing the use of medications and investigated those studies as a subgroup. One previous meta-analysis assessing the association between CRP, IL-6 and depression employed more relaxed inclusion criteria and sample material which may partially explain the smaller overall effect size estimates described in that study (CRP, *d* = 0.15 and IL-6, *d* = 0.25) (Howren et al., 2009). The meta-analysis conducted by Hiles et al. (2012b) found that the association between IL-6 and depression did not significantly differ between smokers and non-smokers and samples analysed by different processing techniques. However, in agreement with our cumulative meta-analysis and previous standard meta-analyses, inclusion of patients using psychotropic medication and the lack of adjustment for overweight/obesity resulted in slightly larger effect size estimates between the levels of CRP, IL-6 and depression (Hiles et al., 2012b; Howren et al., 2009).

The observed heterogeneity between study-specific effect estimates was either high (TNF-α and IL-1β) or moderate (IL-6 and CRP). While some of the subgroup analyses, e.g. studies including older subjects or patients with moderate depression (IL-1β), younger participants (CRP) or more men (IL-6) were associated with reduced overall heterogeneity between studies, none of these factors significantly affected the overall associations. On the other hand, heterogeneity remained high (*I*^2^ > 79%) in all additional analyses examining the relationship between TNF-α and major depression. These results indicate that some other undefined factors may be modifying the observed associations. For example, genetic variations and brain abnormalities have been proposed to be associated with depression-related immune activation (Bufalino et al., 2013; Savitz et al., 2013) and developmental influences, such as childhood trauma, may also be linked to increased vulnerability to develop psychiatric disorders in later life via inflammatory processes (Tursich et al., 2014). Furthermore, it is possible that inflammation is only linked to some specific characteristic symptoms of major depression. Exploration of the role of gene-environment interactions and other potential moderators affecting on the strength and the nature of immune–depression relationships remains a challenge for future investigators.

The limitations of the current study need to be taken into account when interpreting the results. First, when determining the co-occurrence of inflammation and major depression, we focused on a cross-sectional association based on one-time assessments of inflammatory markers, although these measurements cannot reliably distinguish between chronic and acute inflammation. The few previous studies assessing chronic inflammation have revealed stronger associations with mental health when inflammation is determined using repeated measurements rather than only one measurement (Gimeno et al., 2009; Kivimaki et al., 2014). If this also applies to the situation with major depression, then the present data may, if anything, underestimate the role of the specific inflammation markers in major depressive disorders. Due to the lack of longitudinal studies using repeated immune assessments to investigate the role of inflammation in clinically depressed patients, the strength of these associations remains uncertain.

Second, the lack of well-designed and adequately controlled studies (none of the included studies received a maximum quality score of 9% and 41% of all studies were scored as low quality, scores 0–5) highlight the need for future studies with improved study designs and more comprehensive control for confounding variables. Third, as only four studies on IL-6 and seven on TNF-α had differentiated data for different subtypes of major depression (e.g. melancholic, atypical, suicidal, recurrent and/or treatment-resistant depression) we could not include a more detailed analysis assessing the association of subtypes of major depressive disorders with circulating inflammatory markers. Fourth, comparison of major depression patients with and without comorbid conditions with healthy controls may inflate the observed group differences because a number of comorbid conditions may increase both inflammation and depression. Therefore, future studies investigating the association between inflammation and major depression should pay more attention to ensure that they control properly for the inflammatory effects of physical conditions.

## 5 Conclusions

Cumulative meta-analyses are important in collating the totality of data, potentially helping researchers and funders to make informed decisions about the sufficiency of evidence and to set priorities for new studies. Ideally, this will contribute to a faster accumulation of knowledge and reduced costs and effort by preventing unnecessary research. Our cumulative meta-analysis of four inflammatory markers sought to bring together the findings from studies on the co-occurrence of elevated systemic levels of interleukins 6 and 1β, tumour necrosis factor α and C-reactive protein and major depressive disorders. The strong evidence of increased circulating concentrations of interleukin-6 and C-reactive protein in patients with major depressive disorder but not on antidepressant medication compared with non-depressed individuals confirms the cross-sectional association between inflammation and major depression. Peripherally administered IL-6 has been shown to cross the blood–brain barrier (Banks et al., 1994), suggesting that immunomodulatory studies aimed at reducing IL-6 in subgroups of patients with an elevated inflammatory profile could have potential in the treatment of patients with depression and enhanced levels of inflammation. While concentrations of TNF-α and IL-1β appeared to be elevated in certain subgroups of patients, the role of confounding factors, such as age and obesity, should be taken into account when planning future research on these immune markers. Furthermore, future studies are needed to monitor symptomatic presentations, classify depressive symptoms and control more comprehensively for confounders potentially interfering with immune–depression relations (including lifestyle factors, other psychiatric disorders, comorbid physical conditions and medications used at the time of assessments). More attention should also be paid to the accuracy of the measurement of IL-1β.

## Funding

This work was supported by the Economic and Social Research Council (J023299), Medical Research Council (K013351 and G1001354), UK and NordForsk, the Nordic Council of Ministers (75021).

## Figures and Tables

**Table 1:** Summary statistics on the associations between levels of CRP, IL-6, TNF-α and IL-1β and major depressive disorder in different subgroups.

	IL-6					CRP					TNF-α					IL-1β				
Studies (*N*)	*d*	*p*	*Q*	*I*^2^	Studies (*N*)	*d*	*p*	*Q*	*I*^2^	Studies (*N*)	*d*	*p*	*Q*	*I*^2^	Studies (*N*)	*d*	*p*	*Q*	*I*^2^
All studies	31	0.54	0.000	68.5	56.2	20	0.46	0.000	50.9	62.3	31	0.40	0.002	253.8	88.2	14	−0.05	0.860	197.2	93.4
Subjects not using antidepressants	22	0.59	0.000	43.8	52.1	10	0.60	0.001	31.5	71.4	19	0.54	0.001	138.5	87.0	12	−0.09	0.758	187.7	94.1
Subjects using medication	10	0.43	0.000	19.2	53.2	10	0.36	0.000	15.3	41.3	13	0.28	0.211	111.1	89.2	3	0.16	0.690	7.60	73.7
Study subjects under 40 years old	13	0.51	0.000	19.7	39.0	10	0.45	0.000	9.19	2.0	16	0.56	0.001	117.9	87.3	9	−0.45	0.205	139.9	94.3
Study subjects over 40 years old	18	0.58	0.000	48.7	65.1	10	0.52	0.003	41.9	78.5	15	0.21	0.300	132.5	89.4	5	0.65	0.000	6.65	39.9
>50% of study subjects female	19	0.53	0.000	46.4	61.2	14	0.52	0.000	38.9	66.5	23	0.40	0.004	175.1	87.4	12	−0.05	0.858	186.7	94.1
<50% of study subjects female	10	0.55	0.000	11.5	22.0	4	0.31	0.255	10.6	71.7	7	0.55	0.122	73.6	91.8	2	0.02	0.979	5.47	81.7
BMI controlled	16	0.48	0.000	36.2	58.5	11	0.39	0.000	11.2	10.8	16	0.25	0.129	123.7	87.9	8	−0.12	0.710	97.7	92.8
BMI not controlled	15	0.62	0.000	30.4	54.0	8	0.55	0.005	35.2	80.1	15	0.57	0.006	128.9	89.1	6	0.04	0.938	89.5	94.4
Study size < 50	14	0.51	0.000	24.6	47.1	9	0.36	0.042	20.4	60.8	11	0.53	0.150	121.5	91.8	4	0.33	0.271	9.07	66.9
Study size ⩾ 50	17	0.56	0.000	43.8	63.5	11	0.53	0.000	29.7	66.4	20	0.32	0.007	123.0	84.5	10	−0.18	0.598	181.0	95.0
Study subjects inpatients	10	0.60	0.000	24.8	63.7	7	0.53	0.012	35.7	83.2	8	0.44	0.017	33.9	79.3	3	−0.19	0.453	7.22	72.3
Study subjects outpatients	18	0.45	0.000	31.4	45.8	10	0.38	0.000	10.4	13.2	19	0.24	0.190	176.4	89.8	10	−0.12	0.748	165.5	94.6
Quality score < 6	10	0.41	0.000	13.2	32.0	10	0.26	0.032	20.0	54.9	13	0.58	0.006	85.3	85.9	5	0.61	0.004	10.5	61.9
Quality score ⩾ 6	21	0.60	0.000	52.6	62.0	10	0.69	0.000	21.7	58.5	18	0.28	0.089	159.3	89.3	9	−0.36	0.291	142.5	94.4
Mild/moderate depression^a^	6	0.46	0.054	21.6	76.9	5	0.37	0.011	4.92	19.7	7	−0.16	0.568	58.9	89.8	3	0.64	0.000	1.25	0.00
Severe depression^b^	21	0.62	0.000	37.7	47.0	13	0.50	0.000	45.4	73.6	22	0.58	0.000	177.2	88.2	11	−0.25	0.431	171.7	94.2

a Mild/moderate depression: score <19 (HAMD), <35 (MADRS), <39 (IDS) or <30 (BDI).

b Severe depression: score ⩾19 (HAMD), ⩾35 (MADRS), ⩾39 (IDS) or ⩾30 (BDI).
