# Supplementary material for: Cumulative meta-analysis of interleukins 6 and 1β, tumour necrosis factor α and C-reactive protein in patients with major depressive disorder
Source: Brain Behav Immun. 2015 Oct;49:206–15. doi: 10.1016/j.bbi.2015.06.001 (PMC4566946; doi:10.1016/j.bbi.2015.06.001)
Supplement: Supplementary data 1 [file mmc1.pdf]

Cumulative meta-analysis of interleukins 6 and 1β, tumour necrosis factor α and C-reactive protein in patients with major depressive disorder

Rita Haapakoski, Julia Mathieu, Klaus P Ebmeier, Harri Alenius, Mika Kivimäki

Supplementary Material

Tables:

Quality assessment of included studies 2

Summary statistics for included studies 4

Inclusion and exclusion criteria in the studies and covariates included in the meta-analysis 7

Comorbid psychiatric conditions, substance use, severity of depression and medications 13

Figures:

Flow diagram on literature search strategy 16

Cumulative meta-analysis on A) IL-6, B) CRP, C) TNF-α and D) IL1β including only studies with high quality (score ≥6) and subjects not using antidepressants 17

References 19

Supplementary Table 1. Quality assessment of studies included in the meta-analysis using Newcastle-Ottawa scale for case-control studies.

Study	*Selection*	*Comparability*	*Exposure*	*Total*
Basterzi AD et al., 2005	**	*	**	5
Carvalho LA et al., 2013	***	**	**	7
Cizza G et al., 2009	***	**	**	7
Dahl J et al., 2014	***	**	***	8
Dhabhar FS et al., 2009	****	**	**	8
Diniz BS et al., 2010a	**	*	*	4
Diniz BS et al., 2010b	**	*	*	4
Dome P et al., 2009	**	**	*	5
Dunjic-Kostic B et al., 2013	**	**	**	6
Eller T 2008	**	*	***	6
Eller T 2009	**	**	**	6
Euteneuer F et al., 2011	**	**	**	6
Fitzgerald P et al., 2006	**	*	**	5
Fornaro M et al., 2011	*	*	**	4
Fornaro M et al., 2013	*	*	***	5
Frodl T et al., 2013	**	*	**	5
Grassi-Oliveira, R et al., 2009	**	**	**	6
Hennings A et al., 2013	****	**	**	8
Hernandez ME et al., 2008	***	**	***	8
Hernandez ME et al., 2013	**	**	**	6
Hornig M et al., 1998	**		*	3
Huang T-L & Lee C-T 2007	*	**	***	6
Hughes MM et al., 2012	***	**	***	8
Häfner S et al., 2008	**		*	3
Joyce PR et al., 1992	*	**	**	5
Kagaya A 2001	**	*	**	5
Karlović D et al., 2012	**	*	***	6
Kéri S et al., 2014	***	*	**	6
Kling MA et al., 2006	***	**	**	7
Lanquillon S et al., 2000	**	**	***	7
Leo R et al., 2006	**	**	***	7
Li Z et al., 2013	**	**	**	6
Maes M et al., 1995a	**	*	***	6
Maes M et al., 1995b	***	*	***	7
Maes M et al., 1997	**	*	***	6
Maes M, Mihaylova I et al., 2012	**	*	**	5
Maes M, Ringel K et al., 2012	**	*	*	4

…continued

Study	*Selection*	*Comparability*	*Exposure*	*Total*
Mikova O et al., 2001	*	*	***	5
Motivala SJ et al., 2005	***	**	**	7
Narita K et al., 2006	**	**	**	6
O'Brien S et al., 2007	**		***	5
O'Donovan A et al., 2013	**	*	***	6
Pavon L et al., 2006	***	**	***	8
Pike JL and Irwin MR 2006	***	**	**	7
Piletz JE et al.,2009	***	*	**	6
Rothermundt M et al., 2001	*	*	***	5
Rudolf S et al., 2014	**	*	**	5
Schmidt FM 2014	**	*	**	5
Simon NM et al., 2008	**	*	**	5
Sluzewska A et al., 1996	**	*	***	6
Sutcigil L et al., 2007	**	*	***	6
Thomas AJ et al., 2005	**	*	*	4
Tuglu C et al., 2003	**	*	**	5
Voderholzer U et al., 2012	**	**	**	6
Weinstein, AA et al., 2010	***	**	**	7
Yang K et al., 2007	**	**	***	7
Yoshimura R et al., 2010	*	*	**	4
Zahn D et al., 2012	*	*	**	4

Quality points for *exposure* assessment were:

- ascertainment of method (blood drawn as fasting state and use of validated assays for sample analysis) = *

- same procedures used for cases and controls (sample processing and assay performance) = *

- the representativeness of sample material (three or more immune markers analysed in the study) = *

Supplementary Table 2. Summary statistics for included studies.

Study	*Patient type*	*Immune measures*	*N (cases/contr.)*	*Gender (female/male)*	*Age (cases/controls)*	*Depression diagnosis and severity scale*	*Country*	*Quality score*
Basterzi AD et al., 2005[1]	Outpatients	IL-6	46 (23/23)	40/6	33.8 ± 12.8 / 33.6 ± 12.5	DSM-IV, HAM-D	Turkey	5
Carvalho LA et al., 2013[2]	Inpatients	IL-6, IL-10, IL-4, MCP-1	40 (19/21)	28/12	49.05 ± 3.3 / 45.9 ± 2.4	DSM-IV, ICD-10, HAM-D	UK	7
Cizza G et al., 2009[3]	Outpatients	CRP	133 (89/44)	women only	35.5 ± 7 / 35.2 ± 7	DSM-IV, HAM-D	USA	7
Dahl J et al., 2014[4]	Outpatients	IL-1β, IL-1Ra, Il-5, IL-6, IL-7, IL-8, IL-10, G-CSF, IFN-γ, MIP-1α, TNF-α, IL-2, IL-15	84 (50/34)	57/27	40 ± 12 / 38.3 ± 13.9	MINI, MADRS, IDS	Norway	8
Dhabhar FS et al., 2009[5]	Outpatients	IL-6, IL-10	23 (12/11)	13/10	38.42 ± 11.03 / 38.00 ± 13.27	DSM-IV, HAM-D, IDS	USA	8
Diniz BS et al., 2010a[6]	Outpatients	IL-1β	67 (23/44)	57/10	70.2 ± 4.9 / 69.5 ± 7.1	DSM-IV, HAM-D	Brazil	4
Diniz BS et al., 2010b[7]	Outpatients	TNF-α, sTNFR1, sTNFR2	67 (28/39)	55/11	70.4 ± 4.38 / 70.2 ± 5.3	DSM-IV, HAM-D	Brazil	4
Dome P et al., 2009[8]	In- and outpatients	CRP, TNF-α	49 (33/16)	43/6	40.6 ± 10.6 / 40.3 ± 9.5	DSM-IV, BDI	Hungary	5
Dunjic-Kostic B et al., 2013[9]	Inpatients	IL-6, TNF-α, sIL-2R	86 (47/39)	38/48	51.27 ± 7.35 / 49.90 ± 4.99	DSM-IV, HAM-D	Serbia	6
Eller T 2008[10]	Outpatients	IL-8, TNF-α, sIL-2R	145 (100/45)	99/48	32.1 ± 11.9 / 32.9 ± 14.1	MINI, MADRS	Estonia	6
Eller T 2009[11]	Outpatients	TNF-α, sIL-2R	130 (75/55)	89/41	35.2 ± 13.7 / 32.75 ± 14.10	MINI, HAM-D, BDI	Estonia	6
Euteneuer F et al., 2011[12]	Outpatients	IL-6, TNF-α	85 (37/48)	51/34	33.46 ± 12.77 / 35.79 ± 13.25	DSM-IV, BDI-II,SCL-90-R	Germany	6
Fitzgerald P et al., 2006[13]	Inpatients	IL-6, TNF-α	38 (19/19)	26/12	46.4 ± 2.92 / 40.1 ± 2.44	DSM-IV, HAM-D	Ireland	5
Fornaro M et al., 2011[14]	Outpatients	IL-6	32 (16/16)	21/11	51.1 ± 11 / 44 ± 11.6	DSM-IV, HAM-D	Italy	4
Fornaro M et al., 2013[15]	Outpatients	IL-1β, TNF-α, IFN-γ, IL-2, IL-4, IL-10, IL-12	62 (30/32)	48/14	48.27 ± 9.67 / 45.23 ± 11.62	DSM-IV, HAM-D	Italy	5
Frodl T et al., 2012[16]	Outpatients	IL-6, CRP	83 (40/43)	49/34	41.4 ± 10.9 /37.0 ± 13.7	DSM-IV, HAM-D, BDI	Ireland	5
Grassi-Oliveira, R et al., 2009[17]	Outpatients	TNF-α, sTNFR1, sTNFR2	49 (30/19)	women only	39.21 ± 8.56 / 37.36 ± 5.48	DSM-IV, BDI	Brazil	6
Hennings A et al., 2013[18]	Outpatients	IL-6	86 (38/48)	24/62	32.08 ± 12.25 / 36.44 ± 13.28	DSM-IV, SOMS7, BDI	Germany	8
Hernandez ME et al., 2008[19]	Outpatients	IL-10, IL-4, IL-13, IL-2, IL-1β, IFN-γ	53 (31/22)	37/16	32.0 ± 9.40 / 30.75 ± 6.25	MINI, HAM-D, BDI	Mexico	8
Hernandez ME et al., 2013[20]	Outpatients	IL-10, IL-4, IL-13, IL-2, IL-1β, IFN-γ	95 (65/30)	69/26	34 ± 9 / 32 ± 6	MINI, HAM-D, BDI	Mexico	6

…continued

Study	Patient type	Immune measures	N (cases/contr.)	Gender (female/male)	Age (cases/controls)	Depression diagnosis and severity scale	Country	Quality score
Hornig M et al., 1998[21]	Outpatients	CRP, TNF-α	68 (22/46)	33/35	44.5 ± 11.6 / 41.1 ± 11.5	DSM-III-R	USA	3
Huang T-L & Lee C-T 2007[22]	Inpatients	IL-1β, TNF-α, IL-10	82 (42/40)	55/27	38.0 ± 8.2 / 31.4 ± 3.9	DSM-IV, HAM-D	Taiwan	6
Hughes MM et al., 2012[23]	Inpatients	IL-6, IL-1β, TNF-α, IFN-γ, CRP	78 (39/39)	45/33	41.9 ± 1.8 / 37.1 ± 2.1	DSM-IV, HAM-D	Ireland	8
Häfner S et al., 2008[24]	In- and outpatients	CRP	138 (70/68)	79/59	49.3 ± 13.3 / 41.3 ± 14.1	DSM-IV, HAM-D	Germany	3
Joyce PR et al., 1992[25]	Outpatients	CRP, AAC, AAT, HAPT, CPM, IgG, IgA, IgM	33 (21/12)	men only	34.2 ± 10.1 / 31.5 ± 4.1	DSM-III-R, HAM-D	New Zealand	5
Kagaya A 2001[26]	Outpatients	IL-6, IL-1β, TNF-α, sIL-2R	23 (6/18)	6/18	31.1 ± 8.2 / 30.9 ± 7.0	DSM-III-R, HAM-D	Japan	5
Karlović D et al., 2012[27]	In- and outpatients	CRP, IL-6, TNF-α	73 (55/18)	30/43	49.75 ± 12.2 / 45.0 ± 9.5	DSM-IV, MINI, HAM-D	Croatia	6
Kéri S et al., 2014[28]	Outpatients	IL-6, CRP	80 (50/30)	52/28	22.6 ± 6.0 / 24.3 ± 8.3	DSM-IV, HAM-D	Hungary	6
Kling MA et al., 2007[29]	Outpatients	CRP, SAA	36 (18/18)	women only	41 ± 12 / 36 ± 10	DSM-IV, HAM-D	USA	7
Lanquillon S et al., 2000[30]	Inpatients	CRP	39 (24/15)	22/13	53.5 ± 15.2 / 48.7 ± 14.1	DSM-IV, HAM-D, MADRS	Germany	7
Leo R et al., 2006[31]	In- and outpatients	IL-6, IL-1β, TNF-α, CD40L, sP-selectin	92 (46/46)	53/39	34.85 ± 5.88 / 34.11 ± 5.22	DSM-IV, HAM-D	Italy	7
Li Z et al., 2013 [32]	In- and Outpatients	TNF-α	128 (64/64)	103/25	32.1 ± 6.8 / 31.6 ± 5.9	DSM-IV, HAM-D	China	6
Maes M et al., 1995a[33]	In- and outpatients	IL-6, sIL-6R,sIL-2R, TfR	99 (61/38)	42/57	36.6 ± 1.3 / 33.8 ± 1.5	DSM-III-R, HAM-D	Belgium	6
Maes M et al., 1995b[34]	Inpatients	IL-6, IL-1β, sIL-2R, TfR	41 (28/13)	16/25	35.2 ± 12.2 / 34.4 ± 15.1	DSM-III-R	USA	7
Maes M et al., 1997[35]	Outpatients	IL-6, IL-6R, IL-1R, CC16, sCD8	50 (35/15)	21/19	50.3 ± 13.9 / 47.5 ± 15.0	DSM-III-R, HAM-D	Belgium	6
Maes M, Mihaylova I et al., 2012[36]	Outpatients	IL-1β, IL-1α, TNF-α	111 (85/26)	64/47	42.0 ± 11.0 / 42.1 ± 12.8	DSM-IV, MINI, HAM-D	Belgium	5
Maes M, Ringel K et al., 2012[37]	Outpatients	IL-1, TNF-α	144 (109/35)	82/62	43.0 ± 11.0 / 42.5 ± 12.4	DSM-IV, HAM-D	Belgium	4
Mikova O et al., 2001[38]	Inpatients	IL-6, IL-8, IL-2R, TNF-α, CC16	43 (28/15)	31/12	47.3 ± 11.3 / 42.0 ± 10.9	DSM-IV, HAM-D	Bulgaria	5
Motivala SJ et al., 2005[39]	Outpatients	IL-6, IL-6sR, MCP-1, sICAM	40 (22/18)	men only	44.4 ± 7.5 / 40.3 ± 9.2	DSM-IV, HAM-D	USA	7
Narita K et al., 2006[40]	Outpatients	TNF-α	41 (21/20)	21/20	60.9± 7.1 / 59.4 ± 4.6	DSM-IV, HAM-D	Japan	6
O'Brien S et al., 2007[41]	Outpatients	IL-6, sIL-6R, TNF-α, IL-8, IL-10	52 (28/24)	33/19	44.15 / 35.58	DSM-IV, HAM-D	Ireland	5
O'Donovan A et al., 2013[42]	Inpatients	IL-6, TNF-α, IL-10, TGF-β, CRP	124 (76/48)	86/36	50.54 ± 2.31 / 45.85 ± 2.23	MINI, HAM-D	Ireland	6

…continued

Study	Patient type	Immune measures	N (cases/contr.)	Gender (female/male)	Age (cases/controls)	Depression diagnosis and severity scale	Country	Quality score
Pavon L et al., 2006[43]	Outpatients	IL-6, TNF-α, IL-1β, IL-2, IFN-γ, IL-4, IL-13	66 (33/33)	56/5	33.6 ± 10.2 / 24.2 ± 1.98	DSM-IV, HAM-D	Mexico	8
Pike JL and Irwin MR 2006[44]	Outpatients	IL-6, IL-2r, haptoglobin, AAT,AAG	50 (25/25)	men only	42.5 ± 9.2 / 42.7 ± 12.0	DSM-IV, HAM-D	USA	7
Piletz JE et al.,2009[45]	Outpatients	CRP, TNF-α, IL-1β, MCP-1, CD40L	39 (22/17)	33/6	39.4 ± 1.9 / 39.7 ± 2.1	DSM-IV, HAM-D	USA	6
Rothermundt M et al., 2001[46]	Inpatients	CRP, A2M, HB	86 (43/43)	56/30	44.45 ± 9.95 / 44.45 ± 9.95	DSM-IV, ICD-10	Germany	5
Rudolf S et al., 2014[47]	Inpatients	CRP, IL-6	56 (32/24)	28/28	34.6 ± 11.7 / 30.8 ± 9.5	DSM-IV	Germany	5
Schmidt FM 2014[48]	In- and outpatients	IL-2, IL-4, IL-5, IL-10, IL-12, IL-13, GM-CSF, IFN-γ, TNF-α	270 (64/206)	163/107	39.09 ± 12.86 / 37.73 ± 12.97	DSM-IV, HAM-D, BDI-II	Germany	5
Simon NM et al., 2008[49]	Outpatients	MCP-1α, MIP-1α, IL-1α, IL-1β, IL-2, IL-3, IL-4, IL-5, IL-6, IL-7, IL-8, IL-10, IL-12p70, IL-13,IL-15, Eotaxin, GM-CSF, IFN-γ, IP-10, TNF-α	98 (49/49)	41/57	41.65 ± 11.07 / 41.69 ± 11.28	DSM-IV	USA	5
Sluzewska A et al., 1996[50]	Inpatients	IL-6, sIL-6, sIL-2R, TfR, CRP, AGP	64 (49/15)	49/15	42.3 ± 6.5 / n/a	DSM-III-R, HAM-D	Poland	6
Sutcigil L et al., 2007[51]	Outpatients	IL-2, IL-4, IL-12, TNF-α, TGF-β, MCP-1	48 (23/25)	23/25	34.78 ± 7.42 / 34.32 ± 7.80	DSM-IV, HAM-D	Turkey	6
Thomas AJ et al., 2005[52]	Outpatients	IL-1β, CRP	40 (21/19)	25/15	76.4 ± 7.3 / 74.9 ± 7.0	DSM-IV, MADRS	UK	4
Tuglu C et al., 2003[53]	Inpatients	CRP, TNF-α	43 (26/17)	17/26	39.38 ± 14.56 / 37.11 ± 11.08	DSM-IV, HAM-D, BDI	Turkey	5
Voderholzer U et al., 2012[54]	Outpatients	IL-6, IL-1RA, IL-2R	33 (16/17)	21/11	34 ± 13 / 27 ± 8	DSM-IV, HAM-D	Germany	6
Weinstein, AA et al., 2010[55]	Outpatients	IL-6, TNF-α, CRP	28 (14/14)	14/14	41.7 ± 9.6 / 39.3 ± 5.6	DSM-IV, HAM-D, BDI-II	USA	7
Yang K et al., 2007[56]	Inpatients	IL-6, TNF-α, IL-1β	46 (23/23)	30/16	42.12 ± 2.27 / 38.39 ± 1.76	DSM-IV, HAM-D	China	7
Yoshimura R et al., 2010[57]	Outpatients	IL-6	40 (20/20)	14/26	39.2 ± 10.6 / 36.7 ± 10.1	DSM-IV, HAM-D	Japan	4
Zahn D et al., 2013[58]	Outpatients	CRP	46 (21/25)	25/21	49.5 ± 8.0 / 54.0 ± 8.4	DSM-IV, QIDS	Germany	4

Diagnostic criteria for depression: DSM: the Diagnostic and Statistical Manual of Mental Disorders; MINI: The Mini International Neuropsychiatric Interview

Depression severity assessment: HAMD: the Hamilton Rating Scale for Depression; MADRS: Montgomery–Åsberg Depression Rating Scale; BDI: the Beck Depression Inventory; ICD: the International Classification of Diseases; IDS: Inventory of Depressive Symptomatology; QIDS: Quick Inventory of Depressive Symptomatology; SCL-90-R: Symptom Checklist-90-Revised

Supplementary Table 3. Inclusion and exclusion criteria in the studies and covariates included in the meta-analysis.

Study	***Inclusion criteria***	***Exclusion criteria***	***Covariates identified and/or controlled in the study***	***Covariates included in the meta-analysis***
Basterzi AD et al., 2005[1]	MDD outpatients	Any other axis I/II DSM-IV diagnosis; current pregnancy; acute or chronic infections; autoimmune, allergic, neoplastic or endocrine diseases; acute physical disorders; surgery; infarction within 3 months	Age; sex; HAMD score	Age; sex; patient type; medications; HAMD score
Carvalho LA et al., 2013[2]	Treatment resistant MDD inpatients; receipt of intensive inpatient treatment; diagnosis of primary affective disorder; failure to respond to at least one medication trial; HAMD score ≥16	Significant physical illnesses; history of hypersensitivity to corticosteroids or steroid use; heavy smoker; use of drugs with known immune/endocrine function: e.g. oral contraceptives; pregnant/lactating women; alcohol dependence	Age; sex; BMI; BDI score; HAMD score; BHS; BAI; RLCQ; BSI; duration of episode; number of previous hospital admissions; ECT in the past; medications	Age; sex; BMI; patient type; BDI score; medications; HAMD score
Cizza G et al., 2009[3]	Female MDD outpatients who had experienced depressive episode in the past three years	Coronary artery disease	Age; BMI; race; smoking; drinking; education; cooper test score; medications; HAMD/HAMA score; age of onset; no of episodes; duration of depression; antidepressant use	Age; BMI, HAMD score; patient type; medications
Dahl J et al., 2014[4]	MDD outpatients with age 18-60 years, score ≥22 on the IDS scale; minimum of 3 weeks washout period	Present or previous hypomania or mania; psychotic symptoms; autoimmune disorder; chronic inflammation; severe metabolic syndrome; BMI>34kg/m²; psychotic disorder; alcohol/drug dependence; receiving anti-inflammatory, antiviral, antibiotic or immune modulating drugs; inability to provide informed consent	Age; sex; BMI; MADRS score; IDS score	Age; sex; BMI; patient type; medications; MADRS score; IDS score
Dhabhar FS et al., 2009[5]	MDD outpatients; HAMD score ≥17; medically healthy; no clinically significant abnormalities; negative urine toxicology; age 19-69 years; English speaking; ability to provide informed consent	Pregnancy; medical illnesses (e.g. autoimmune disease, diabetes, HIV, endocrine disorders, hepatitis, cancer, chronic infections); use of steroids, psychotropic meds, antioxidants, corticosteroids, contraceptives, immunotherapy or antibiotics; febrile illness or elevated WBC counts; immunizations within 4 weeks; DSM-IV criteria for psychotic, bipolar or PTSD; drug/alcohol abuse	Age; sex; BMI; IDS score; HAMD score; marital status; ethnicity; education	Age; sex; BMI; patient type; IDS score; HAMD score; medications
Diniz BS et al., 2010a[6]	Elderly MDD outpatients (first or recurrent episode)	History of disorders of chronic inflammatory disorders or uncontrolled clinical diseases; antidepressant or anti-inflammatory medication	Age; sex; education; depression episode duration; MMSE; CAMCOG; CDT; VF; HAMD score	Age; sex; patient type; HAMD score; medications
Diniz BS et al., 2010b[7]	Elderly MDD outpatients (first or recurrent episode)	History of disorders of chronic inflammatory disorders or uncontrolled clinical diseases; antidepressant or anti-inflammatory medication	Age; sex; education; depression episode duration; MMSE; CAMCOG; CDT; VF; HAMD score	Age; sex; patient type; HAMD score; mediactions
Dome P et al., 2009[8]	MDD in- and outpatients	Elevated levels of fasting blood glucose, creatinine or urea nitrogen or liver functions; hypertension; psychiatric diagnoses; BMI >30kg/m², high cholesterol or triglyceride levels; hypertriglyceridemia; signs of infection; >20mm h erythrocyte sedimentation rate; cardiovascular risk factors or CVD; medications with known effects on EPC numbers	Age; sex; BMI; smoking; BUN; WBC count; total cholesterol; triglycerides; BDI score; medications	Age; sex; BMI; BDI score; medications

…continued

Study	*Inclusion criteria*	*Exclusion criteria*	*Covariates identified and/or controlled in the study*	*Covariates included in the meta-analysis*
Dunjic-Kostic B et al., 2013[9]	MDD inpatients; HAMD score ≥17; examination and diagnosis for the same type of depression; refractory patients	History of any other psychiatric disorder e.g. schizophrenia, other psychosis or substance/alcohol abuse, OCD or organic mental disorder; acute or chronic physical disease; pregnancy or breast feeding; history of systemic, endocrine or immune disorder; cancer; infections; allergies; obesity; under-nutrition; heavy smoker; anti-inflammatory or immunosuppressive therapy	Age; sex; BMI; smoking; marital state; education; employment; age of onset; duration of depression; subtype of depression; HAMD score; duration and class of antidepressant treatment; heredity; suicidal risk	Age; sex; BMI; patient type; HAMD score; medications
Eller T 2008[10]	MDD Outpatients; score ≥23 in MADRS	Acute infections; neurological or immunological disorders; substance abuse; bipolar depression; panic disorder	Age; sex; number of episodes; age of onset; duration of episode; subtype of depression; drug naïve vs. previously treated; MADRS score	Age; sex; patient type; MADRS score; medications
Eller T 2009[11]	MDD outpatients; HAMD score <20; physically healthy patients not receiving physical therapy	Substance abuse; acute infections; neurological or immunological disorders; bipolar disorder; panic disorder; antidepressant or anti-inflammatory medication	Age; sex; BMI; smoking; subtype of depression; HAMD score; BDI score	Age; sex; BMI; patient type; HAMD score; BDI score; medications
Euteneuer F et al., 2011[12]	MDD outpatients	Organic illnesses involving CNS or affecting immune status; psychotic symptoms; somatization or pain disorder; alcohol/drug abuse; analgesics; antipsychotics; stimulants; anxiolytics; current psychotherapy; pregnancy and lactation	Age; sex; BMI; physical activity; years of education; contraceptives; anxiety disorders comorbidity; antidepressants; BDI score; SCL-90-R GSI; pressure pain threshold	Age; sex; BMI; BDI score; patient type; medications
Fitzgerald P et al., 2006[13]	Physically healthy subjects with MDD and without psychotic features; age 25-65 years	Other psychiatric disorders ; inflammatory or allergic conditions; recent or current use of corticosteroids; concurrent physical illness	Age; sex; class and dose of antidepressant medications; HAMD score; severity of depression; SIV score	Age; sex; patient type; HAMD score; medications
Fornaro M et al., 2011[14]	Outpatients diagnosed with single or recurrent, drug naïve MDD episode; age 18-65 years; able to give informed consent; HAMD score ≥18	Pregnancy; breast feeding; bipolar disorder; any anxiety or schizoaffective disorder; schizophrenia; dementia; current suicidal and/or psychotic ideation; other non-psychotropic medications; history of medical comorbidities	Age; sex; HAMD score; YMRS	Age; sex; patient type; HAMD score; medications
Fornaro M et al., 2013[15]	MDD outpatients diagnosed with single or recurrent, drug naïve MDD episode; age 18-65 years; able to give informed consent; HAMD score ≥18	Pregnancy; breast feeding; bipolar disorder; any anxiety or schizoaffective disorder; schizophrenia; dementia; current suicidal and/or psychotic ideation; substance use disorder; other non-psychotropic medications; history of medical comorbidities	Age; sex; HAMD score; YMRS	Age; sex; patient type; HAMD score; medications
Frodl T et al., 2012[16]	MDD outpatients	Antipsychotics or mood stabilizers; age <18 or >65; history of neurological or comorbid psychiatric Axis I/II disorders; severe medical illness; head injury; substance abuse	Age; sex; height; weight; drinking; HAMD score; BDI score; CTQ; childhood maltreatment; age of onset; duration of depression; medications, GILZ; SGK-1; GR	Age; sex; patient type; BDI score; HAMD score; medications
Grassi-Oliveira, R et al., 2009[17]	Female MDD outpatients; 20-55 years of age	Axis I comorbidities; severe or unstable clinical illness; illness associated with abnormal immunological parameters; neurological disorder; psychotic symptoms; any psychoactive substance use in the last 30 days	Age; BMI; education; social status; PCL-C scores; BDI score; PTSD; PCL-C; CTQ; antidepressants	Age; BMI; BDI score; patient type; medications
Hennings A et al., 2013[18]	MDD outpatients with a maximum of three persistent medically unexplained bodily symptoms	Current delusional disorders; alcohol/substance abuse or dependence; persistent medical illnesses that could affect immune status; ongoing psychotherapy; medical illnesses or injuries in the last two week; medication with opiates	Age; sex; BMI; BDI score; fitness status; chronic stress; antidepressants; comorbid Axis I disorder	Age; sex; BMI; patient type; BDI score; medications

…continued

Study	Inclusion criteria	Exclusion criteria	Covariates identified and/or controlled in the study	Covariates included in the meta-analysis
Hernandez ME et al., 2008[19]	MDD outpatients with BMI≤25; low coffee, alcohol and tobacco intake; free of antidepressants for at least 3 weeks	Pregnancy; medical illnesses; pharmacological treatments; history of allergies or allergic reactions; mental disorders including symptoms of dysthymia or chronic depression	Age; sex; BMI; HAMD score; BDI score; education; family history of depression; first or recurrent depression episode	Age; sex; BMI; patient type; HAMD score; BDI score; medications
Hernandez ME et al., 2013[20]	MDD outpatients who were free of antidepressants for at least 3 weeks	Medical illnesses	Age; sex; BMI; HAMD score; BDI score; education; family history of depression; first or recurrent depression episode	Age; sex; BMI; patient type; HAMD score; medications
Hornig M et al., 1998[21]	MDD outpatients	Inflammatory, infectious or allergic diseases in the past two weeks; free of drugs known to affect immune function for four weeks; chronic medical illnesses known to affect immune system	Age; sex; affective subtypes; medications	Age; sex; ; patient type; medications
Huang T-L & Lee C-T 2007[22]	MDD inpatients without any medication two weeks before the study	Chronic medical illnesses including heart, lung, liver, kidney and metabolic diseases; free of acute infections or allergic reactions	Age; sex; BMI; duration of illness; HAMD score	Age; sex; BMI; patient type; HAMD score; medications
Hughes MM et al., 2012[23]	MDD inpatients	Age <18 or >65; history of neurological or comorbid psychiatric disorders (Axis I or Axis II); other severe medical illness; head injury; substance abuse; antipsychotics or mood stabilizers	Age; sex; BMI; smoking; drinking; age of onset; depression duration; HAMD score; antidepressants	Age; sex; BMI; patient type; HAMD score; medications
Häfner S et al., 2008[24]	In- and outpatients with acute MDD	Severe medical disorders and infections; antihypertensive medication such as ACE inhibitors or angiotensin receptor blockers; hormone replacement therapy	Age; sex; BMI; smoking; HAMD score; ACE; hyperlipidemia	Age; sex; BMI; HAMD score; medications
Joyce PR et al., 1992[25]	Male MDD outpatients free of medication for a minimum of two weeks	Physical illnesses	Age; BMI; mean cortisol; delta TSH; HAMD score	Age; BMI; patient type; HAMD score; medications
Kagaya A 2001[26]	MDD outpatients free of antidepressants for more than 1 week at the beginning of the study	Other psychiatric disorders; endocrine and other medical disturbances; bipolar disorders; manic episodes; history of treatment with electroconvulsive therapy or lithium carbonate	Age; sex; HAMD score	Age; sex; patient type; HAMD score; medications
Karlović D et al., 2012[27]	MDD in- and outpatients without psychotropic medication for at least 30 days prior the study	Other psychiatric disorders; alcohol or substance abuse; past and chronic illnesses or any other medical problems	Age; sex; smoking; education; marital status; working status; place of living; severity and subtypes of depression; number of depression episodes; duration of the disorder; psychiatric familiarity; age of onset; HAMD score	Age; sex; HAMD score; medications
Kéri S et al., 2014[28]	First-episode MDD outpatients without a history of previous treatment and not receiving any pharmacological treatment or psychotherapy	Presence and history of psychotic or manic symptoms; severe suicidality requiring emergency treatment; substance misuse in the past 6 months; general health problems requiring medications; inability to participate in the psychotherapeutic process	Age; sex; education; socioeconomic status; smoking; drinking; BMI; waist-to-hip ratio; oral contraceptives; HAMD score	Age; sex; BMI; patient type; HAMD score; medications
Kling MA et al., 2007[29]	Female MDD outpatients with at least 2 previous episodes and off psychotropic medication for ≥3 months	Potentially confounding illnesses; pregnancy; lactating women; smokers; medication for treatment or for prevention of medical condition	Age; BMI; ethnicity; HAMD score	Age; BMI; patient type; HAMD score; medications
Lanquillon S et al., 2000[30]	MDD inpatients free of antidepressant medication for at least 6 weeks before hospital admission	Negative serum and urine screens for antidepressants and substances typical of drug abuse; other Axis I or Axis II diagnosis; pregnancy; acute or chronic infectious, autoimmune, allergic, neoplastic or endocrine diseases; other acute physical diseases	Age; sex; number of depression episodes; duration of current episode; weight loss; HAMD score, MADRS score	Age; sex; patient type; HAMD score; medications

…continued

Study	Inclusion criteria	Exclusion criteria	Covariates identified and/or controlled in the study	Covariates included in the meta-analysis
Leo R et al., 2006[31]	First-episode MDD in- and outpatients not using psychotropic medication	Medical or neurological diseases; other psychiatric disorders; conventional risk factors for CAD incl. hypertension, hyperlipidemia, obesity, diabetes, family history and smoking; use of steroids, NSAIDs or antibiotics during the 2 preceding weeks; contraceptive hormonal therapy	Age; sex; BMI; total cholesterol; LDL cholesterol; triglycerides; HAMD score	Age; sex; BMI; HAMD score; medications
Li Z et al., 2013 [32]	First-episode and drug nave MDD in- and outpatients; age 18-60	HAMD score <17; smoking, drinking or substance dependence; sever medical illness such as cancer, diabetes); brain disease; pregnancy; infectious, auto-immunological, endocrinological, pulmonary, cardiac or haematological disease	Age; sex; BMI; duration of depression; HAMD score	Age; sex; BMI; HAMD score; medications
Maes M et al., 1995a[33]	MDD in- and outpatients	Other Axis I diagnosis; e.g. substance use disorder; organic mental disorder, schizophrenia; abnormal SGPT, SGOT, GGT, hematologic measures and renal function test; drugs known to interfere with immune or endocrine function; chronic illnesses known to affect immune status; acute infectious or allergic reactions for at least 2 weeks before the study	Age; sex; length of wash-out period	Age; sex; medications
Maes M et al., 1995b[34]	MDD inpatients in acute phase of illness and free from antidepressants for at least 1 week before the study	Medical illnesses	Age; sex; smoking;	Age; sex; patient type; medications
Maes M et al., 1997[35]	MDD inpatients	Medical illnesses; infections; inflammatory or allergic reactions for at least 2 weeks before blood sampling; drugs known to affect immune or endocrine function; glucocorticoids; anticonceptive drugs; other Axis I diagnoses (organic mental disorders; schizophrenia; primary anxiety disorders) ; psychoactive substance use disorders within past 6 months; eating disorders	Age: sex; number of depressive episodes; duration of depression; HAMD score	Age: sex; patient type; HAMD score; medications
Maes M, Mihaylova I et al., 2012[36]	MDD outpatients	Medical illnesses; mood stabilizers or immunomodulatory drugs; alcohol abuse and smoking; inflammatory of allergic reactions two months prior study; BMI>30; current and lifetime diagnosis of other Axis I disorders (psychotic, and lifetime diagnoses of anxiety disorders)	Age: sex; depression subtype; number of depressive episodes; duration of depression; HAMD score; use of antidepressants; treatment resistance; CF	Age: sex; HAMD score; patient type; medications
Maes M, Ringel K et al., 2012[37]	MDD outpatients	Medical illnesses; mood stabilizers or immunomodulatory drugs; alcohol abuse and smoking; inflammatory of allergic reactions two months prior study; BMI>30; current and lifetime diagnosis of other Axis I disorders e.g. psychotic, substance use disorders, organic and substance use disorders	Age: sex; depression duration and subtype; number of depressive episodes; HAMD score; use of antidepressants; lysozyme; CF; 5-HT autoimmunity	Age: sex; HAMD score; patient type; medications
Mikova O et al., 2001[38]	MDD inpatients; HAMD score ≥18	Physical illness; MAO inhibitors, antipsychotics, lithium, anticonvulsants during the last 6 months; antidepressants; treated for MS with clucocorticoids during the month before the study; infection; allergic responses for 2 weeks prior study	Age; sex; HAMD score	Age; sex; patient type; HAMD score; medications
Motivala SJ et al., 2005[39]	Male MDD outpatients	Current or history of alcohol dependence; recent viral infections; chronic medical conditions such as diabetes, cancer or COPD; hypertension; antihypertensive medication; antidepressants; aspirin use for 7 days before immune assessment; HIV negativity	Age; weight; ethnicity; education; smoking; drinking; HAMD score; EEG sleep variables	Age; BMI; patient type; HAMD score; medications
Narita K et al., 2006[40]	MDD outpatients with complete remission and receiving antidepressant treatment over 6 months	History of chronic inflammatory disease; collagen disease; cardiac disease; atherosclerotic risk factors; diabetes; chronic alcoholism; smoking; BMI >26; use of antipsychotics, statins or antihypertensive drugs	Age; sex; BMI; fasting glucose; HbA1c; number and duration of depression; duration of pharmacotherapy; adiponectin; SSRI/SNRI; HAMD score	Age: sex; BMI; HAMD score; patient type; medications

…continued

Study	Inclusion criteria	Exclusion criteria	Covariates identified and/or controlled in the study	Covariates included in the meta-analysis
O'Brien S et al., 2007[41]	MDD outpatients with HAMD score >20	Significant physical illness including acute or chronic infections, inflammatory or immune disorders; >10% above ideal body weight; endocrine, immune or metabolic disorder; IBD; acquired immunodeficiency syndrome; allergic, infectious or inflammatory response in last 2 weeks; other Axis I disorders; substance abuse	Age; sex; smoking; HAMD score	Age; sex; HAMD score; medications
O'Donovan A et al., 2013[42]	MDD inpatients	Lack of primary diagnosis of MDD; acute physical illness; age <18 years	Age; sex; BMI; HAMD score, suicidal ideation; attempted suicide; number of depression episodes; duration of MDD; medications	Age; sex; BMI; HAMD score, patient type; medications
Pavon L et al., 2006[43]	MDD outpatients free of medication for at least three weeks; HAMD score ≥22	Past and present neurological, psychiatric or substance abuse; physical illnesses; no comorbid diagnoses besides anxiety; low coffee, tobacco and alcohol consumption	Age; sex; ethnicity; socio-economic class; education	Age; sex; patient type; medications
Pike JL and Irwin MR 2006[44]	MDD male outpatients in good health and not using antidepressants for at least 19 days prior to evaluation	Alcohol or substance abuse or dependence; psychiatric comorbidity; recent <10 days viral illness; immunosuppressive medications;	Age; weight; smoking; drinking; HAMD score; medications	Age; sex; patient type; HAMD score; medications
Piletz JE et al.,2009[45]	MDD outpatients with wash-out period for at least 2 weeks; HAMD score ≥19	Medical illness such as CAD, diabetes, arthritis or hypertension; any diagnosis other than MDD; clinically significant abnormalities; history of seizures; pregnancy; allergy to antidepressants; abnormality on ECG; history of substance abuse/dependence; PTSD; psychosis; bleeding diathesis; history of heart or vascular disease; family history of early-onset heart or vascular disease; heavy smoking (>1 pack of cigarettes/day)	Age; sex; BMI; systolic and diastolic BP; HAMD score; medications	Age; sex; BMI; HAMD score; patient type; medications
Rothermundt M et al., 2001[46]	MDD inpatients	Acute infectious diseases; past physical illnesses e.g. acute or chronic infections, autoimmune diseases, cancer); medications with potential immune effects	Age; sex; HAMD score; depression subtype; medications	Age; sex; HAMD score; patient type; medications
Rudolf S et al., 2014[47]	MDD inpatients	Schizophrenia; mental retardation; alcohol or drug abuse for at least 3 months prior to the study; diabetes; inflammatory of infectious disease; CVD; pregnancy; age ≤17 years; polypharmacy	Age; sex; height; weight; BMI; sport; alcohol; pack years; waist girth; systolic/diastolic BP; cholesterol; HDL; LDL; triglycerides; HbA1c; glucose; insulin; cortisol; depression subtype; medications	Age; sex; BMI; patient type; medications
Schmidt FM 2014[48]	MDD in- and outpatients free of psychoactive medication for at least 7 days prior the examination	Other DSM-IV Axis I disorders; use of illegal drugs: alcohol abuse within the past 6 months; history of head injury with loss of consciousness >1h; acute or chronic infection; major somatic disorders; diagnosed sleep apnea; obesity hypoventilation syndrome	Age; sex; BMI; smoking; employment; activity; age at onset; duration of depression; number of episodes; duration of current episode; sleep patterns; appetite; HAMD score; BDI score;	Age; sex; BMI; HAMD score; BDI score; patient type; medications
Simon NM et al., 2008[49]	MDD outpatients free of antidepressants for a minimum of 1 week at entry	Pregnancy; significant medical or substance use disorder	Age; sex; ethnicity; duration of depression; HAMD score	Age; sex; HAMD score; patient type; medications
Sluzewska A et al., 1996[50]	MDD inpatients who were drug-free for at least 10 days	Chronic or acute infection; allergic disease or other condition affecting immune system for at least 2 weeks before the study; major medical illness; oral contraceptives	Age; sex; HAMD score	Age; sex; HAMD score; patient type; medications

…continued

Study	Inclusion criteria	Exclusion criteria	Covariates identified and/or controlled in the study	Covariates included in the meta-analysis
Sutcigil L et al., 2007[51]	First episode MDD outpatients	Other Axis I or Axis II diagnosis; pregnancy; acute or chronic infections within the past month; autoimmune, allergic, neoplastic or endocrine diseases and other acute physical disorders within past 3 months; use of antidepressants, NSAIDs or oral contraceptives in the past 6 weeks	Sex; age; HAMD score	Sex; age; HAMD score; patient type; medications
Thomas AJ et al., 2005[52]	>60 years old MDD outpatients with score ≥20 on the MADRS	Dementia or other neurological diseases; conditions associated with inflammation such as rheumatoid arthritis; oral steroids	Age; sex; cognitive performance; MARDS; mini-mental state examination; geriatric depression scale; the age at onset; duration of depression; medications	Age; sex; MADRS; patient type; medications
Tuglu C et al., 2003[53]	MDD inpatients without antidepressive medications for the last 6 weeks	Other Axis I or Axis II DSM-IV diagnosis; pregnancy; chronic or acute disease in the last 3 months; neuroleptics (past 6 months)	Age; sex; HAMD score; BDI score; ESR: leukocytes	Age; sex; HAMD score; BDI score; patient type; medications
Voderholzer U et al., 2012[54]	Drug-free MDD outpatients	Significant medical disorder or infectious disease; smoking; history of drug/alcohol abuse; psychotropic or anti-inflammatory drugs within 7 days; restless leg syndrome, sleep apnea	Age; sex; BMI; HAMD score; TSD	Age; sex; BMI; patient type; HAMD score; medications
Weinstein, AA et al., 2010[55]	MDD outpatients	Age <18 or >80 years; history of CAD; antihypertensive, immunomodulatory or anti-inflammatory medications other than aspirin; current or past diagnosis or treatment of bipolar disorder or psychosis; active suicidal ideation	Age; sex; BMI; race; smoking; activity level; mental stress; HAMD score; medications	Age; sex; BMI; HAMD score; patient type; medications
Yang K et al., 2007[56]	MDD inpatients with HAMD score ≥20 and free of major psychotropic drugs for at least 4 weeks (fluoxetine ≥5 weeks)	Significant physical or neurological illness; acute or chronic infections; inflammatory and immune disorders; aspirin and NSAIDs for at least 2 weeks; therapeutic corticosteroids for at least 6 months prior testings; schizophrenia or other psychoses; organic mental disorders; substance dependence; OCD or any other primary psychiatric disorder	Age; sex; BMI; education; menostasis; age of onset; duration of illness and current episode; number of episodes; family history; HAMD score	Age; sex; BMI; HAMD score; patient type; medications
Yoshimura R et al., 2010[57]	MDD outpatients without any medications for at least 2 weeks before the blood samplings	Physical illnesses; alcohol/drug abuse;	Age; sex; HAMD score; duration of the illness; age of onset; plasma BDNF levels	Age; sex; BMI; HAMD score; patient type; medications
Zahn D et al., 2013[58]	Untreated MDD outpatients	History of coronary heart, cerebrovascular of peripheral artery disease; current substance abuse; psychotic illness; suicidal tendencies; use of platelet aggregation inhibitors; anticoagulant or antidepressant medication; cognitive impairment; severe medical conditions other than hypertension and dyslipidaemia	Age; sex; BMI; education; smoking; HbA1c; HDL; LDL; triglycerides; CD40; quick inventory of depressive symptomology (QIDS); medications	Age; sex; BMI; patient type; medications

Abbreviations: ACE: angiotensin-converting enzyme; ACTH: adrenocorticotropic hormone; BAI: Beck anxiety inventory; BDI: Beck depression inventory; BDNF: brain-derived neurotropic factor; BHS: Beck hopelessness scale; BMI: body mass index; BP: blood pressure; BSI: Beck suicide scale; BUN: blood urea nitrogen; CAMCOG: Cambridge cognitive examination; CDT: clock drawing test; CF: chronic fatigue; COPD: chronic obstructive pulmonary disease; CTQ: childhood trauma questionnaire; CVD: cardiovascular disease; ECG: electrocardiogram; ECT: electroconvulsotherapy; EEG: electroencephalography; EPC: endothelial progenitor cells; ESR: erythrocyte sedimentation rate; GGT: The gamma-glutamyl transferase ; GILZ: glucocorticoid-inducible genes Leucine Zipper; GR: glucocorticoid receptor; GSI: global severity index: HAMA: Hamilton anxiety scale; HAMD: Hamilton depression scale; HbA1c: glycosylated haemoglobin A1c; HDL: high-density lipoprotein; 5-HT: 5-hydroxytryptamine; IBD: inflammatory bowel disease; IDS: inventory of depressive symptomology; LDL: low-density lipoprotein; MADRS: Montgomery Åsberg depression scale; MAOI: monoamine oxidase inhibitor; MMSE: mini mental state examination; NSAID: non-steroidal anti-inflammatory drug; OCD: obsessive-compulsive disorder; PCL-C: PTSD checklist-civilian version; PTSD: post-traumatic stress disorder; RLCQ: recent life changes questionnaire; SCL: symptom check list; SGK-1: serum and glucocorticoid-inducible kinase-1; SGOT: serum glutamic oxaloacetic transaminase; SGPT: serum glutamic pyruvic transaminase; SIV: steroid-induced vasoconstriction; TSD: total sleep deprivation; TSH: thyroid stimulating hormone; SSRI: selective serotonin reuptake inhibitor; SRNI: serotonin–norepinephrine reuptake inhibitors; VF: verbal fluency; WBC: white blood cells; YMRS: young mania rating scale.

Supplementary Table 4. Other Axis I or Axis II disorders, subtypes and the severity of depression, substance use and medications..

Study	***Any other Axis I or Axis II disorder****	***Depression subtype provided****	***Depression severity informed***$^{\delta}$	***Substance use~***	***Medications not used at the time of assessment₸***	***Wash-out period time (min)***	***Medications used at the time of assessment^***	***Medication use (Yes/No category)***
Basterzi AD et al., 2005[1]	No	n/a	Yes (S)	n/a	1, 2, 6, 7	6wk	n/a	No
Carvalho LA et al., 2013[2]	n/a	Yes 4 (100%)	Yes (S)	No (1, 2)	7	n/a	1, 2, 3, 4, 5, 6	Yes
Cizza G et al., 2009[3]	Yes 1B (50%)	n/a	Yes (M)	Yes (1)	n/a	n/a	2, 7	Yes
Dahl J et al., 2014[4]	No (2, 3)	Yes 1 (76%)	Yes (M)	No (2, 3)	1, 2, 6, 8, 11	3wk	n/a	No
Dhabhar FS et al., 2009[5]	No (2, 3, 4)	n/a	Yes (S)	No (2, 3)	1, 2, 3, 5, 7, 8, 9	6wk	n/a	No
Diniz BS et al., 2010a[6]	n/a	n/a	Yes (M)	n/a	1, 2, 6	n/a	n/a	No
Diniz BS et al., 2010b[7]	n/a	n/a	Yes (S)	n/a	1, 2, 6	n/a	n/a	No
Dome P et al., 2009[8]	No	n/a	Yes (S)	Yes (1)	n/a	n/a	1, 2, 3, 4, 5, 6, 10	Yes
Dunjic-Kostic B et al., 2013[9]	No (3, 5, 7)	Yes 1 (62%) 2 (38%)	Yes (S)	Yes (1) No (2, 3)	1, 2, 3, 4, 5, 6, 9	4wk	n/a	No
Eller T 2008[10]	No (2, 6)	Yes 1 (73%)	Yes (M)	No (2, 3)	6	n/a	1, 7, 11	Yes
Eller T 2009[11]	No (2, 6)	Yes 1 (85%)	Yes (S)	Yes (1) No (2, 3)	1, 2, 6, 9	2wk	7	No
Euteneuer F et al., 2011[12]	Yes 1A (5.3%), 6 (8.1%), 4 (10.5%), 8 (18.5%) No 3	n/a	Yes (M)	No (2, 3)	4, 5, 6, 11	n/a	1, 2	Yes
Fitzgerald P et al., 2006[13]	No (3)	Yes (4)	Yes (S)	n/a	9	n/a	1, 2	Yes
Fornaro M et al., 2011[14]	No (1B, 2, 5)	n/a	Yes (S)	n/a	1, 2, 6, 7	15d	4, 11 (sedative)	No
Fornaro M et al., 2013[15]	No (1B, 2, 5)	n/a	Yes (S)	No (2, 3)	1, 2, 6, 7	15d	4, 11 (sedative)	No
Frodl T et al., 2013[16]	No	n/a	Yes (S)	No (2, 3)	3, 5	n/a	1, 2	Yes
Grassi-Oliveira, R et al., 2009[17]	No (3) Yes (4)	n/a	Yes (M)	Yes (1)	5	n/a	1, 2	Yes
Hennings A et al., 2013[18]	Yes (57.9%) No (3)	n/a	Yes (M)	No (2, 3)	11 (opiates)	n/a	1, 2	Yes
Hernandez ME et al., 2008[19]	No	n/a	Yes (S)	Yes (1) No (2)	1, 2, 5	3wk	n/a	No
Hernandez ME et al., 2013[20]	n/a	n/a	Yes (S)	n/a	1, 2	3wk	n/a	No
Hornig M et al., 1998[21]	No (2)	n/a	n/a	n/a	n/a	n/a	1, 2, 4, 5	Yes

…continued

Study	*Any other Axis I or Axis II disorder**	*Depression subtype provided*•	*Depression severity informed*δ	*Substance use~*	*Medications not used at the time of assessment*₸	*Wash-out period time (min)*	*Medications used at the time of assessment^*	*Medication use (Yes/No category)*
Huang T-L & Lee C-T 2007[22]	n/a	Yes 1 (60%) 3 (26%)	Yes (S)	n/a	Subjects “medication free”	2wk	n/a	No
Hughes MM et al., 2012[23]	No	n/a	Yes (S)	Yes (1) No (2)	2	n/a	1, 2	Yes/No
Häfner S et al., 2008[24]	n/a	n/a	Yes (S)	n/a	10	n/a	1, 2	Yes
Joyce PR et al., 1992[25]	n/a	n/a	Yes (S)	n/a	1, 11 (anticonvulsants)	2wk	n/a	No
Kagaya A 2001[26]	No (2)	n/a	Yes (S)	n/a	1, 2	1wk	n/a	**No**
Karlović D et al., 2012[27]	No	Yes 1 (58%) 2 (42%)	Yes (S)	Yes (1) No (2, 3)	1, 2, 4	30d	n/a	No
Kéri S et al., 2014[28]	No (2, 3)	n/a	Yes (S)	Yes (1) No (2)	1, 2, 3	n/a	7	No
Kling MA et al., 2007[29]	n/a	n/a	Yes (M)	No (1)	1, 2	3mo	n/a	No
Lanquillon S et al., 2000[30]	No	n/a	Yes (S)	No (2, 3)	1, 2	6wk	n/a	No
Leo R et al., 2006[31]	No	n/a	Yes (S)	No (1)	1, 2, 4, 6, 7, 8	2wk	n/a	No
Li Z et al., 2013[32]	n/a	Yes 3 (70%)	Yes (S)	No (2, 3)	1, 2	n/a	n/a	No
Maes M et al., 1995a[33]	No (5)	n/a	n/a	No (2, 3)	1, 2	1wk	n/a	No
Maes M et al., 1995b[34]	No (5)	n/a	n/a	Yes (1)	1, 2, 5	1wk	n/a	No
Maes M et al., 1997[35]	No (1B, 5)	Yes 4 (80%)	Yes (S)	No (3)	2, 7, 9	n/a	n/a	No
Maes M, Mihaylova I et al., 2012[36]	No (1B, 3)	Yes 1 (68%), 4 (68%)	Yes (S)	No (1, 2)	2, 3, 6, 9, 10	n/a	n/a	Yes/No
Maes M, Ringel K et al., 2012[37]	No (3)	Yes 1 (83%), 4 (54%)	Yes (S)	No (1, 2, 3)	9, 10	n/a	1, 2	Yes
Mikova O et al., 2001[38]	No	n/a	n/a	n/a	1, 2, 3, 10	n/a	n/a	No
Motivala SJ et al., 2005[39]	n/a	n/a	Yes (S)	Yes (1) No (2)	1, 2, 4, 10, 11 (aspirin)	n/a	n/a	No
Narita K et al., 2006[40]	n/a	No	Yes (M)	No (1, 2)	5, 10, 11 (statins)	n/a	1, 2, 4	Yes
O'Brien S et al., 2007[41]	No	n/a	Yes (S)	Yes (1) No (2, 3)	n/a	n/a	1, 2, 11 (lithium)	Yes
O'Donovan A et al., 2013[42]	n/a	Yes 3 (38%)	Yes (S)	n/a	n/a	n/a	6, 11 (statins)	Yes
Pavon L et al., 2006[43]	Yes (1B)	n/a	Yes (S)	Yes (1) No (2, 3)	Subjects “medication free”	3wk	n/a	No
Pike JL and Irwin MR 2006[44]	No	n/a	Yes (S)	Yes (1) No (2, 3)	1, 2, 4	7d	9, 11 (diuretic, β-agonist)	No

…continued

Study	*Any other Axis I or Axis II disorder**	*Depression subtype provided*⁎	*Depression severity informed*ᵟ	*Substance use~*	*Medications not used at the time of assessment*₸	*Wash-out period time (min)*	*Medications used at the time of assessment^*	*Medication use (Yes/No category)*
Piletz JE et al.,2009[45]	No (3, 4)	n/a	Yes (S)	Yes (1) No (2, 3)	1, 2 (6, 10)	2wk (3d)	n/a	No
Rothermundt M et al., 2001[46]	No (3)	Yes 1 (51%)	Yes (S)	Yes (1)	n/a	n/a	1, 2	Yes
Rudolf S et al., 2014[47]	No (5)	Yes 2 (25%)	n/a	Yes (1) No (2, 3)	2, 4	n/a	1	Yes
Schmidt FM 2014[48]	No	n/a	Yes (M)	Yes (1) No (2, 3)	1, 2, 4	7d	n/a	No
Simon NM et al., 2008[49]	Yes 1B (48%) 6 (15%) 9 (35%) 4 (18%)	n/a	Yes (S)	Yes 2, 3 (35%)	1, 2	1wk	(6, not assessed)	No
Sluzewska A et al., 1996[50]	n/a	n/a	Yes (S)	n/a	7 (subjects "medication free")	10d	n/a	No
Sutcigil L et al., 2007[51]	No	n/a	Yes (S)	n/a	1, 2, 6, 7	6wk	n/a	No
Thomas AJ et al., 2005[52]	n/a	n/a	Yes (M)	Yes (1)	11 (steroids)	n/a	1, 2, 4, 6	Yes
Tuglu C et al., 2003[53]	No	n/a	Yes (S)	n/a	1, 2, 11 (neuroleptics)	6wk	n/a	No
Voderholzer U et al., 2012[54]	n/a	n/a	Yes (M)	Yes(1) No (2, 3)	1, 2, 4, 6	7d	7	No
Weinstein, AA et al., 2010[55]	No (2, 3)	n/a	Yes (M)	Yes (1)	6, 10	n/a	1, 2, aspirin	Yes
Yang K et al., 2007[56]	No (3, 5, 7)	n/a	Yes (S)	No	1, 2, 4, 6, 9	2wk	4 (benzodiazepines)	No
Yoshimura R et al., 2010[57]	n/a	n/a	Yes (M)	No (2, 3)	Subjects "medication free")	2wk	n/a	No
Zahn D et al., 2013[58]	No (3)	n/a	Yes (M)	Yes (1) No (2, 3)	1, 2, 11 (anticoagulant)	n/a	n/a	No

* 1A: generalized anxiety disorder, 1B: " anxiety disorder" 2: bipolar disorder, 3: "psychotic disorder" or "psychoses", 4: post-traumatic stress disorder, 5: schizophrenia, 6: panic disorder, 7: obsessive-compulsive disorder, 8: phobia, 9: social anxiety

⁎ 1 = melancholic, 2 = atypical, 3 = suicidal, 4 = treatment resistant depression

~ 1: smoking, 2: alcohol and/or substance dependence, 3: drug abuse

ᵟ Depression severity (*mean score of all patients*): M = mild/moderate, S = severe (HAMD: mild/moderate: score <19, severe: ≥19; MADR: mild/moderate: <34, severe: >34; IDS: mild/moderate: <39, severe: ≥39; BDI: mild/moderate: <30, severe: ≥30

₸ 1: SSRIs, 2: other antidepressant or class n/a, 3: mood stabilizers, 4: anxiolytics, 5: antipsychotics, 6: anti-inflammatory, 7: contraceptives, 8: antibiotics, 9: corticosteroids, 10: antihypertensive, 11: other

^ 1: SSRIs, 2: other antidepressants or class n/a, 3: mood stabilizers, 4: anxiolytics, 5: antipsychotics, 6: anti-inflammatory, 7: contraceptives, 8: antibiotics, 9: corticosteroids, 10: antihypertensive, 11:other

Supplementary Figure 1. Flow diagram on literature search strategy.

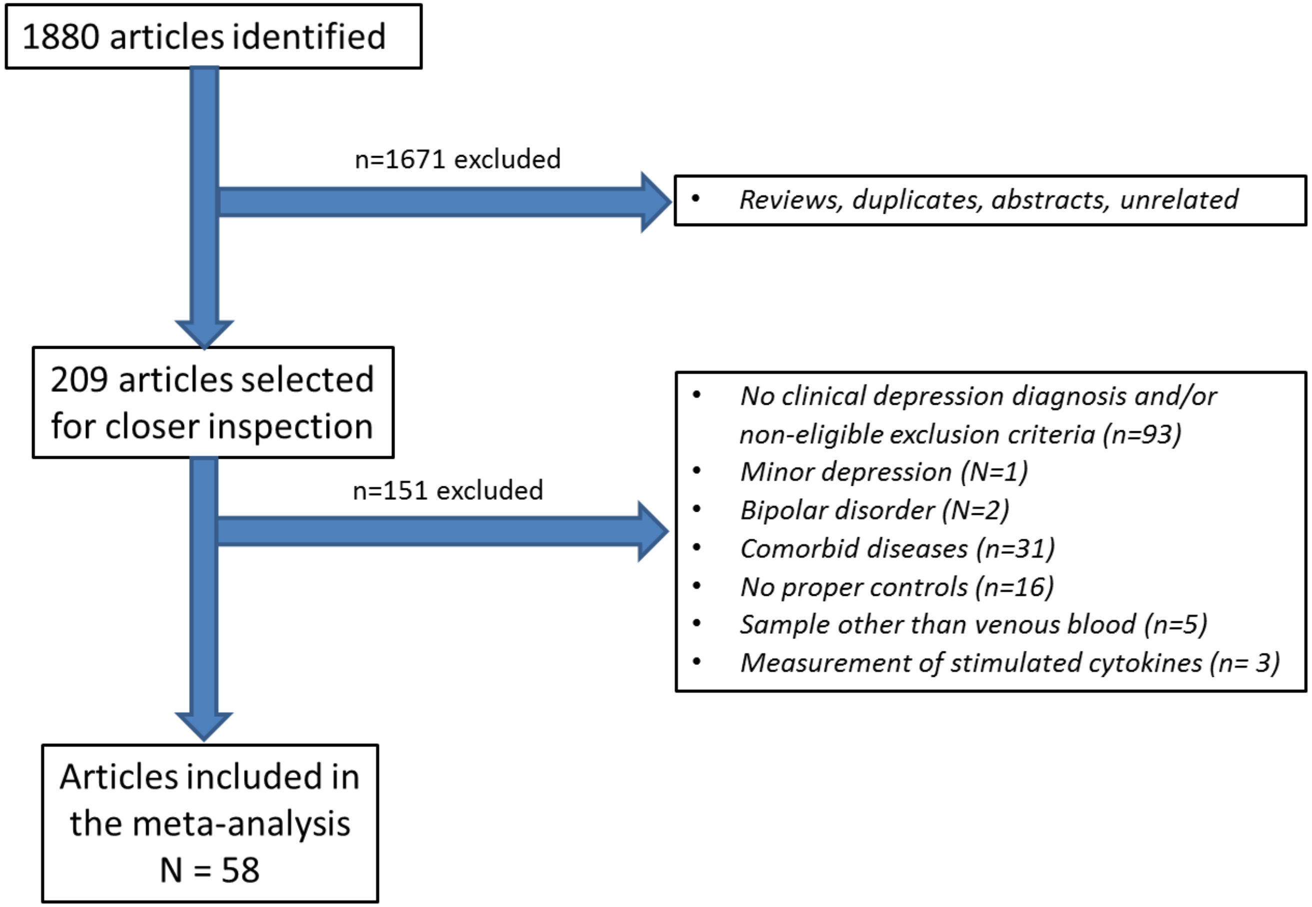

Supplementary Figure 2. Sensitivity analysis: cumulative meta-analysis on A) IL-6, B) CRP, C) TNF-α and D) IL1β including only studies with high quality (score ≥6) and subjects not using antidepressants.

A) IL-6

Study name	Subgroup within study	Point	Standard error	Variance	Lower limit	Upper limit	Z-Value	p-Value	Depressed	Control
		Cumulative statistics								
Maes M et al., 1995a	MDD (all)	0.448	0.200	0.040	0.055	0.841	2.236	0.025	77	38
Maes M et al., 1995b	MDD (all)	0.439	0.172	0.030	0.100	0.777	2.542	0.011	90	66
Sluzewska A et al., 1996	MDD (all)	0.923	0.473	0.224	-0.005	1.851	1.949	0.051	139	81
Maes M et al., 1997	MDD (all)	0.868	0.338	0.114	0.206	1.530	2.570	0.010	174	96
Motivala SJ et al., 2005	MDD (all)	0.859	0.267	0.072	0.335	1.383	3.211	0.001	196	114
Leo R et al., 2006	MDD (all)	0.918	0.224	0.050	0.480	1.356	4.105	0.000	242	160
Pavon L et al., 2006	MDD (all)	0.819	0.207	0.043	0.414	1.224	3.961	0.000	275	193
Pike JL and Irwin MR 2006	MDD (all)	0.807	0.180	0.032	0.453	1.160	4.476	0.000	300	218
Yang K et al., 2007	MDD (all)	0.777	0.160	0.026	0.462	1.091	4.841	0.000	333	241
Dhabhar FS et al., 2009	MDD (all)	0.753	0.150	0.023	0.459	1.047	5.021	0.000	345	252
Hughes MM et al., 2012	no antidepr. meds	0.729	0.141	0.020	0.452	1.006	5.159	0.000	357	264
Karlovic D et al., 2012	Combined	0.734	0.126	0.016	0.487	0.980	5.834	0.000	412	300
Voderholzer U et al., 2012	MDD (all)	0.664	0.135	0.018	0.399	0.930	4.906	0.000	425	315
Dunjic-Kostic B et al., 2013	Combined	0.664	0.121	0.015	0.427	0.901	5.493	0.000	472	393
Dahl J et al., 2014	MDD (all)	0.653	0.111	0.012	0.435	0.871	5.870	0.000	522	427
Kéri S et al., 2014	MDD (all)	0.652	0.103	0.011	0.450	0.854	6.336	0.000	572	457
		0.652	0.103	0.011	0.450	0.854	6.336	0.000		

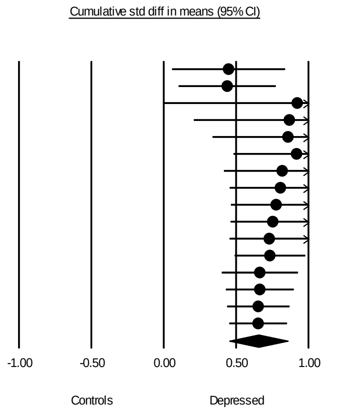

B) CRP

Study name	Subgroup within study	Point	Standard error	Variance	Lower limit	Upper limit	Z-Value	p-Value	Depressed	Control
		Cumulative statistics								
Sluzewska A et al., 1996	MDD (all)	1.453	0.322	0.104	0.822	2.083	4.514	0.000	49	15
Lanquillon S et al., 2000	MDD (all)	1.506	0.244	0.060	1.028	1.985	6.171	0.000	73	30
Kling MA et al., 2006	MDD (all)	1.292	0.220	0.048	0.862	1.723	5.886	0.000	91	48
Piletz JE et al.,2009	MDD (all)	1.058	0.280	0.079	0.508	1.607	3.773	0.000	113	65
Hughes MM et al., 2012	MDD (all)	0.971	0.231	0.054	0.518	1.425	4.199	0.000	125	104
Karlovic D et al., 2012	Combined	0.951	0.176	0.031	0.606	1.296	5.410	0.000	180	140
Kéri S et al., 2014	MDD (all)	0.876	0.158	0.025	0.567	1.185	5.549	0.000	230	170
		0.876	0.158	0.025	0.567	1.185	5.549	0.000		

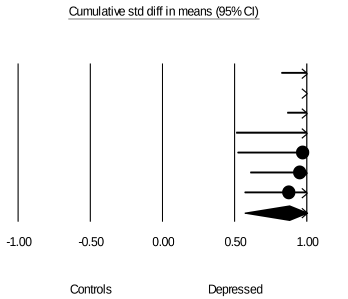

C) TNF-α

Study name	Subgroup within study	Point	Standard error	Variance	Lower limit	Upper limit	Z-Value	p-Value	Depressed	Control
Leo R et al., 2006	MDD (all)	1.334	0.231	0.053	0.882	1.786	5.787	0.000	46	46
Pavon L et al., 2006	MDD (all)	0.970	0.371	0.137	0.244	1.697	2.617	0.009	79	79
Huang T-L & Lee C-T 2007	Combined	0.668	0.376	0.142	-0.070	1.406	1.774	0.076	121	159
Sutcigil L et al., 2007	MDD (all)	1.176	0.491	0.241	0.214	2.138	2.395	0.017	144	184
Yang K et al., 2007	MDD (all)	1.055	0.387	0.150	0.297	1.814	2.728	0.006	177	207
Eller T et al., 2009	Combined	0.818	0.372	0.138	0.089	1.547	2.198	0.028	252	317
Piletz JE et al.,2009	MDD (all)	0.803	0.330	0.109	0.157	1.449	2.436	0.015	274	334
Hughes MM et al., 2012	W/o meds	0.755	0.300	0.090	0.167	1.344	2.518	0.012	286	346
Karlovic D et al., 2012	Combined	0.713	0.259	0.067	0.205	1.222	2.752	0.006	341	382
Dunjic-Kostic B et al., 2013	Combined	0.596	0.246	0.061	0.114	1.078	2.422	0.015	388	460
Li Z et al., 2013	MDD (all)	0.590	0.218	0.048	0.163	1.018	2.706	0.007	452	524
Dahl J et al., 2014	MDD (all)	0.573	0.199	0.039	0.183	0.962	2.883	0.004	502	558
		0.573	0.199	0.039	0.183	0.962	2.883	0.004		

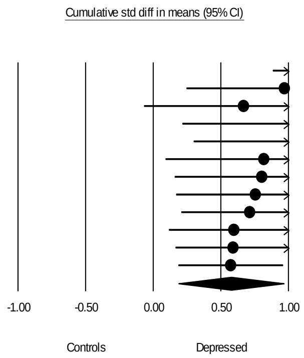

D) IL-1-β

Study name	Subgroup within study	Point	Standard error	Variance	Lower limit	Upper limit	Z-Value	p-Value	Depressed	Control
Leo R et al., 2006	MDD (all)	0.985	0.221	0.049	0.552	1.418	4.462	0.000	46	46
Pavon L et al., 2006	MDD (all)	-0.710	1.703	2.901	-4.048	2.628	-0.417	0.677	79	79
Huang T-L & Lee C-T 2007	Combined	-0.678	0.873	0.763	-2.390	1.034	-0.776	0.438	121	159
Yang K et al., 2007	MDD (all)	-0.472	0.643	0.413	-1.732	0.787	-0.735	0.462	154	182
Hernandez ME et al., 2008	MDD (all)	-0.628	0.546	0.298	-1.697	0.441	-1.151	0.250	185	204
Piletz JE et al.,2009	MDD (all)	-0.404	0.488	0.238	-1.361	0.552	-0.828	0.408	207	221
Hughes MM et al., 2012	Combined	-0.342	0.404	0.163	-1.135	0.450	-0.847	0.397	246	260
Hernandez ME et al., 2013	Combined	-0.469	0.375	0.141	-1.205	0.266	-1.251	0.211	311	320
Dahl J et al., 2014	MDD (all)	-0.363	0.344	0.118	-1.038	0.311	-1.056	0.291	361	354
		-0.363	0.344	0.118	-1.038	0.311	-1.056	0.291		

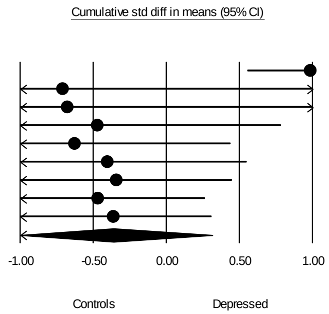

References

1. Basterzi AD, Aydemir C, Kisa C, Aksaray S, Tuzer V, Yazici K, Goka E. IL-6 levels decrease with SSRI treatment in patients with major depression. *Hum Psychopharmacol.* 2005;20(7):473-476.
2. Carvalho LA, Torre JP, Papadopoulos AS, Poon L, Juruena MF, Markopoulou K, Cleare AJ, Pariante CM. Lack of clinical therapeutic benefit of antidepressants is associated overall activation of the inflammatory system. *J Affect Disord.* 2013;148(1):136-140.
3. Cizza G, Eskandari F, Coyle M, Krishnamurthy P, Wright EC, Mistry S, Csako G, Group POWERS. Plasma CRP levels in premenopausal women with major depression: a 12-month controlled study. *Horm Metab Res.* 2009;41(8):641-648.
4. Dahl J, Ormstad H, Aass HC, Malt UF, Bendz LT, Sandvik L, Brundin L, Andreassen OA. The plasma levels of various cytokines are increased during ongoing depression and are reduced to normal levels after recovery. *Psychoneuroendocrinology.* 2014;45:77-86.
5. Dhabhar FS, Burke HM, Epel ES, Mellon SH, Rosser R, Reus VI, Wolkowitz OM. Low serum IL-10 concentrations and loss of regulatory association between IL-6 and IL-10 in adults with major depression. *J Psychiatr Res.* 2009;43(11):962-969.
6. Diniz BS, Teixeira AL, Talib L, Gattaz WF, Forlenza OV. Interleukin-1beta serum levels is increased in antidepressant-free elderly depressed patients. *Am J Geriatr Psychiatry.* 2010;18(2):172-176.
7. Diniz BS, Teixeira AL, Talib LL, Mendonca VA, Gattaz WF, Forlenza OV. Increased soluble TNF receptor 2 in antidepressant-free patients with late-life depression. *J Psychiatr Res.* 2010;44(14):917-920.
8. Dome P, Teleki Z, Rihmer Z, Peter L, Dobos J, Kenessey I, Tovari J, Timar J, Paku S, Kovacs G, Dome B. Circulating endothelial progenitor cells and depression: a possible novel link between heart and soul. *Mol Psychiatry.* 2009;14(5):523-531.
9. Dunjic-Kostic B, Ivkovic M, Radonjic NV, Petronijevic ND, Pantovic M, Damjanovic A, Poznanovic ST, Jovanovic A, Nikolic T, Jasovic-Gasic M. Melancholic and atypical major depression--connection between cytokines, psychopathology and treatment. *Prog Neuropsychopharmacol Biol Psychiatry.* 2013;43:1-6.
10. Eller T, Vasar V, Shlik J, Maron E. Pro-inflammatory cytokines and treatment response to escitalopram in major depressive disorder. *Prog Neuropsychopharmacol Biol Psychiatry.* 2008;32(2):445-450.
11. Eller T, Aluoja A, Maron E, Vasar V. Soluble interleukin-2 receptor and tumor necrosis factor levels in depressed patients in Estonia. *Medicina (Kaunas).* 2009;45(12):971-977.
12. Euteneuer F, Schwarz MJ, Hennings A, Riemer S, Stapf T, Selberdinger V, Rief W. Depression, cytokines and experimental pain: evidence for sex-related association patterns. *J Affect Disord.* 2011;131(1-3):143-149.
13. Fitzgerald P, O'Brien SM, Scully P, Rijkers K, Scott LV, Dinan TG. Cutaneous glucocorticoid receptor sensitivity and pro-inflammatory cytokine levels in antidepressant-resistant depression. *Psychol Med.* 2006;36(1):37-43.
14. Fornaro M, Martino M, Battaglia F, Colicchio S, Perugi G. Increase in IL-6 levels among major depressive disorder patients after a 6-week treatment with duloxetine 60 mg/day: a preliminary observation. *Neuropsychiatr Dis Treat.* 2011;7:51-56.
15. Fornaro M, Rocchi G, Escelsior A, Contini P, Martino M. Might different cytokine trends in depressed patients receiving duloxetine indicate differential biological backgrounds. *J Affect Disord.* 2013;145(3):300-307.
16. Frodl T, Carballedo A, Hughes MM, Saleh K, Fagan A, Skokauskas N, McLoughlin DM, Meaney J, O'Keane V, Connor TJ. Reduced expression of glucocorticoid-inducible genes GILZ and SGK-1: high IL-6 levels are associated with reduced hippocampal volumes in major depressive disorder. *Transl Psychiatry.* 2012;2:e88.

17. Grassi-Oliveira R, Brietzke E, Pezzi JC, Lopes RP, Teixeira AL, Bauer ME. Increased soluble tumor necrosis factor-alpha receptors in patients with major depressive disorder. *Psychiatry Clin Neurosci.* 2009;63(2):202-208.
18. Hennings A, Schwarz MJ, Riemer S, Stapf TM, Selberdinger VB, Rief W. Exercise affects symptom severity but not biological measures in depression and somatization - results on IL-6, neopterin, tryptophan, kynurenine and 5-HIAA. *Psychiatry Res.* 2013;210(3):925-933.
19. Hernandez ME, Mendieta D, Martinez-Fong D, Loria F, Moreno J, Estrada I, Bojalil R, Pavon L. Variations in circulating cytokine levels during 52 week course of treatment with SSRI for major depressive disorder. *Eur Neuropsychopharmacol.* 2008;18(12):917-924.
20. Hernandez ME, Mendieta D, Perez-Tapia M, Bojalil R, Estrada-Garcia I, Estrada-Parra S, Pavon L. Effect of selective serotonin reuptake inhibitors and immunomodulator on cytokines levels: an alternative therapy for patients with major depressive disorder. *Clin Dev Immunol.* 2013;2013:267871.
21. Hornig M, Goodman DB, Kamoun M, Amsterdam JD. Positive and negative acute phase proteins in affective subtypes. *J Affect Disord.* 1998;49(1):9-18.
22. Huang TL, Lee CT. T-helper 1/T-helper 2 cytokine imbalance and clinical phenotypes of acute-phase major depression. *Psychiatry Clin Neurosci.* 2007;61(4):415-420.
23. Hughes MM, Carballedo A, McLoughlin DM, Amico F, Harkin A, Frodl T, Connor TJ. Tryptophan depletion in depressed patients occurs independent of kynurenine pathway activation. *Brain Behav Immun.* 2012;26(6):979-987.
24. Häfner S, Baghai T, Eser D, Schule C, Rupprecht R, Bondy B. C-reactive protein is associated with polymorphisms of the angiotensin-converting enzyme gene in major depressed patients. *Journal of Psychiatric Research.* 2008;42:163-165.
25. Joyce PR, Hawes CR, Mulder RT, Sellman JD, Wilson DA, Boswell DR. Elevated levels of acute phase plasma proteins in major depression. *Biol Psychiatry.* 1992;32(11):1035-1041.
26. Kagaya A, Kugaya A, Takebayashi M, Fukue-Saeki M, Saeki T, Yamawaki S, Uchitomi Y. Plasma concentrations of interleukin-1beta, interleukin-6, soluble interleukin-2 receptor and tumor necrosis factor alpha of depressed patients in Japan. *Neuropsychobiology.* 2001;43(2):59-62.
27. Karlovic D, Serretti A, Vrkic N, Martinac M, Marcinko D. Serum concentrations of CRP, IL-6, TNF-alpha and cortisol in major depressive disorder with melancholic or atypical features. *Psychiatry Res.* 2012;198(1):74-80.
28. Keri S, Szabo C, Kelemen O. Expression of Toll-Like Receptors in peripheral blood mononuclear cells and response to cognitive-behavioral therapy in major depressive disorder. *Brain Behav Immun.* 2014.
29. Kling MA, Alesci S, Csako G, Costello R, Luckenbaugh DA, Bonne O, Duncko R, Drevets WC, Manji HK, Charney DS, Gold PW, Neumeister A. Sustained low-grade pro-inflammatory state in unmedicated, remitted women with major depressive disorder as evidenced by elevated serum levels of the acute phase proteins C-reactive protein and serum amyloid A. *Biol Psychiatry.* 2007;62(4):309-313.
30. Lanquillon S, Krieg JC, Bening-Abu-Shach U, Vedder H. Cytokine production and treatment response in major depressive disorder. *Neuropsychopharmacology.* 2000;22(4):370-379.
31. Leo R, Di Lorenzo G, Tesauro M, Razzini C, Forleo GB, Chiricolo G, Cola C, Zanasi M, Troisi A, Siracusano A, Lauro R, Romeo F. Association between enhanced soluble CD40 ligand and proinflammatory and prothrombotic states in major depressive disorder: pilot observations on the effects of selective serotonin reuptake inhibitor therapy. *J Clin Psychiatry.* 2006;67(11):1760-1766.
32. Li Z, Qi D, Chen J, Zhang C, Yi Z, Yuan C, Wang Z, Hong W, Yu S, Cui D, Fang Y. Venlafaxine inhibits the upregulation of plasma tumor necrosis factor-alpha (TNF-alpha) in the Chinese patients with major depressive disorder: a prospective longitudinal study. *Psychoneuroendocrinology.* 2013;38(1):107-114.

33. Maes M, Meltzer HY, Bosmans E, Bergmans R, Vandoolaeghe E, Ranjan R, Desnyder R. Increased plasma concentrations of interleukin-6, soluble interleukin-6, soluble interleukin-2 and transferrin receptor in major depression. *J Affect Disord.* 1995;34(4):301-309.
34. Maes M, Meltzer HY, Buckley P, Bosmans E. Plasma-soluble interleukin-2 and transferrin receptor in schizophrenia and major depression. *Eur Arch Psychiatry Clin Neurosci.* 1995;244(6):325-329.
35. Maes M, Bosmans E, De Jongh R, Kenis G, Vandoolaeghe E, Neels H. Increased serum IL-6 and IL-1 receptor antagonist concentrations in major depression and treatment resistant depression. *Cytokine.* 1997;9(11):853-858.
36. Maes M, Mihaylova I, Kubera M, Ringel K. Activation of cell-mediated immunity in depression: association with inflammation, melancholia, clinical staging and the fatigue and somatic symptom cluster of depression. *Prog Neuropsychopharmacol Biol Psychiatry.* 2012;36(1):169-175.
37. Maes M, Ringel K, Kubera M, Berk M, Rybakowski J. Increased autoimmune activity against 5-HT: a key component of depression that is associated with inflammation and activation of cell-mediated immunity, and with severity and staging of depression. *J Affect Disord.* 2012;136(3):386-392.
38. Mikova O, Yakimova R, Bosmans E, Kenis G, Maes M. Increased serum tumor necrosis factor alpha concentrations in major depression and multiple sclerosis. *Eur Neuropsychopharmacol.* 2001;11(3):203-208.
39. Motivala SJ, Sarfatti A, Olmos L, Irwin MR. Inflammatory markers and sleep disturbance in major depression. *Psychosom Med.* 2005;67(2):187-194.
40. Narita K, Murata T, Takahashi T, Kosaka H, Omata N, Wada Y. Plasma levels of adiponectin and tumor necrosis factor-alpha in patients with remitted major depression receiving long-term maintenance antidepressant therapy. *Prog Neuropsychopharmacol Biol Psychiatry.* 2006;30(6):1159-1162.
41. O'Brien SM, Scully P, Fitzgerald P, Scott LV, Dinan TG. Plasma cytokine profiles in depressed patients who fail to respond to selective serotonin reuptake inhibitor therapy. *J Psychiatr Res.* 2007;41(3-4):326-331.
42. O'Donovan A, Rush G, Hoatam G, Hughes BM, McCrohan A, Kelleher C, O'Farrelly C, Malone KM. Suicidal ideation is associated with elevated inflammation in patients with major depressive disorder. *Depress Anxiety.* 2013;30(4):307-314.
43. Pavon L, Sandoval-Lopez G, Eugenia Hernandez M, Loria F, Estrada I, Perez M, Moreno J, Avila U, Leff P, Anton B, Heinze G. Th2 cytokine response in Major Depressive Disorder patients before treatment. *J Neuroimmunol.* 2006;172(1-2):156-165.
44. Pike JL, Irwin MR. Dissociation of inflammatory markers and natural killer cell activity in major depressive disorder. *Brain Behav Immun.* 2006;20(2):169-174.
45. Piletz JE, Halaris A, Iqbal O, Hoppensteadt D, Fareed J, Zhu H, Sinacore J, Devane CL. Pro-inflammatory biomakers in depression: treatment with venlafaxine. *World J Biol Psychiatry.* 2009;10(4):313-323.
46. Rothermundt M, Arolt V, Peters M, Gutbrodt H, Fenker J, Kersting A, Kirchner H. Inflammatory markers in major depression and melancholia. *J Affect Disord.* 2001;63(1-3):93-102.
47. Rudolf S, Greggersen W, Kahl KG, Huppe M, Schweiger U. Elevated IL-6 levels in patients with atypical depression but not in patients with typical depression. *Psychiatry Res.* 2014;217(1-2):34-38.
48. Schmidt FM, Lichtblau N, Minkwitz J, Chittka T, Thormann J, Kirkby KC, Sander C, Mergl R, Fasshauer M, Stumvoll M, Holdt LM, Teupser D, Hegerl U, Himmerich H. Cytokine levels in depressed and non-depressed subjects, and masking effects of obesity. *J Psychiatr Res.* 2014;55:29-34.
49. Simon NM, McNamara K, Chow CW, Maser RS, Papakostas GI, Pollack MH, Nierenberg AA, Fava M, Wong KK. A detailed examination of cytokine abnormalities in Major Depressive Disorder. *Eur Neuropsychopharmacol.* 2008;18(3):230-233.

50. Sluzewska A, Rybakowski J, Bosmans E, Sobieska M, Berghmans R, Maes M, Wiktorowicz K. Indicators of immune activation in major depression. *Psychiatry Res.* 1996;64(3):161-167.
51. Sutcigil L, Oktenli C, Musabak U, Bozkurt A, Cansever A, Uzun O, Sanisoglu SY, Yesilova Z, Ozmenler N, Ozsahin A, Sengul A. Pro- and anti-inflammatory cytokine balance in major depression: effect of sertraline therapy. *Clin Dev Immunol.* 2007;2007:76396.
52. Thomas AJ, Davis S, Morris C, Jackson E, Harrison R, O'Brien JT. Increase in interleukin-1beta in late-life depression. *Am J Psychiatry.* 2005;162(1):175-177.
53. Tuglu C, Kara SH, Caliyurt O, Vardar E, Abay E. Increased serum tumor necrosis factor-alpha levels and treatment response in major depressive disorder. *Psychopharmacology (Berl).* 2003;170(4):429-433.
54. Voderholzer U, Fiebich BL, Dersch R, Feige B, Piosczyk H, Kopasz M, Riemann D, Lieb K. Effects of sleep deprivation on nocturnal cytokine concentrations in depressed patients and healthy control subjects. *J Neuropsychiatry Clin Neurosci.* 2012;24(3):354-366.
55. Weinstein AA, Deuster PA, Francis JL, Bonsall RW, Tracy RP, Kop WJ. Neurohormonal and inflammatory hyper-responsiveness to acute mental stress in depression. *Biol Psychol.* 2010;84(2):228-234.
56. Yang K, Xie G, Zhang Z, Wang C, Li W, Zhou W, Tang Y. Levels of serum interleukin (IL)-6, IL-1beta, tumour necrosis factor-alpha and leptin and their correlation in depression. *Aust N Z J Psychiatry.* 2007;41(3):266-273.
57. Yoshimura R, Umene-Nakano W, Hoshuyama T, Ikenouchi-Sugita A, Hori H, Katsuki A, Hayashi K, Atake K, Nakamura J. Plasma levels of brain-derived neurotrophic factor and interleukin-6 in patients with dysthymic disorder: comparison with age- and sex-matched major depressed patients and healthy controls. *Hum Psychopharmacol.* 2010;25(7-8):566-569.
58. Zahn D, Petrak F, Uhl I, Juckel G, Neubauer H, Hagele AK, Wiltfang J, Herpertz S. New pathways of increased cardiovascular risk in depression: a pilot study on the association of high-sensitivity C-reactive protein with pro-atherosclerotic markers in patients with depression. *J Affect Disord.* 2013;146(3):420-425.
